# Metabolic therapies inhibit tumor growth *in vivo* and *in silico*

**DOI:** 10.1038/s41598-019-39109-1

**Published:** 2019-02-28

**Authors:** Jorgelindo da Veiga Moreira, Minoo Hamraz, Mohammad Abolhassani, Laurent Schwartz, Mario Jolicœur, Sabine Peres

**Affiliations:** 1 Research Laboratory in Applied Metabolic Engineering, Department of Chemical Engineering, École Polytechnique de Montréal, P.O. Box 6079, Centre-ville Station, Montréal, Québec Canada; 2 LRI, Université Paris-Sud, CNRS, Université Paris-Saclay, 91405 Orsay, France; 3 MaIAGE, INRA, Université Paris-Saclay, 78350 Jouy-en-Josas, France; 4 Institut Cochin, Université Paris- Descartes, 75014 Paris, France; 5 Nosco Pharmaceuticals, 75015 Paris, France; 6 Assistance Publique des Hôpitaux de Paris, 149 avenue Victoria, 75004 Paris, France

## Abstract

In the recent years, cancer research succeeded with sensitive detection methods, targeted drug delivery systems, and the identification of a large set of genes differently expressed. However, although most therapies are still based on antimitotic agents, which are causing wide secondary effects, there is an increasing interest for metabolic therapies that can minimize side effects. In the early 20^th^ century, Otto Warburg revealed that cancer cells rely on the cytoplasmic fermentation of glucose to lactic acid for energy synthesis (called “Warburg effect”). Our investigations aim to reverse this effect in reprogramming cancer cells’ metabolism. In this work, we present a metabolic therapy specifically targeting the activity of specific enzymes of central carbon metabolism, combining the METABLOC bi-therapeutic drugs combination (Alpha Lipoic Acid and Hydroxycitrate) to Metformin and Diclofenac, for treating tumors implanted in mice. Furthermore, a dynamic metabolic model describing central carbon metabolism as well as fluxes targeted by the drugs allowed to simulate tumors progression in both treated and non-treated mice, in addition to draw hypotheses on the effects of the drugs on tumor cells metabolism. Our model predicts metabolic therapies-induced reversed Warburg effect on tumor cells.

## Introduction

Is cancer a genetic disease or a metabolic alteration? This issue has been subject to debates in the history of cancer^1,2^. The genomic hypothesis of cancer originally raised with the discovery of a proto-oncogene tyrosine-protein kinase (Src) responsible of cancer in chicken. J. Michael Bishop and Harold E. Varmus, two Nobel Prize winners (1989) discovered in 1979 that mutations in *src* gene in normal chickens can lead to cancer^3^. Then, the genomic area of cancer reached its peak with the complet sequencing of the human genome by the International Human Genome Sequencing Consortium in 2001^4^. This has opened new markets for the pharmaceutical industry while defining new approaches in cancer therapy. For instance synthetic drugs such as Cisplatin and 5-Fluorouracil where introduced and are now part of the chemotherapeutic regimen. However, the limited success of these chemotherapeutic agents opened avenues for new strategies. New thoughts wave has emerged with a disruptive synthetic review aiming at redefining “The hallmarks of Cancer” and the common rules that govern the reprogramming of normal cells into malignant cancers^5^. Hanahan and Weinberg described common molecular machinery involved in regulating cell proliferation, differentiation and death. Indeed, dysfunctions on the internal “machine” or on its environment lead to the same deleterious phenotype: “sustaining proliferative signaling, evading growth suppressors, resisting cell death, replicative immortality, sustained angiogenesis, and activating invasion and metastasis”. More recently, the same authors proposed an updated version of the previous review where they added “two emerging hallmarks”, including “reprogramming of energy metabolism and evading immune destruction^6^”. These new concepts have been the subject of numerous studies these last years^7^, including those from our research group^8–11^.

To the question of whether cancer is a genetic disease or a metabolic alteration, hypothesis enabled explaining how cancer cells’ metabolic reprogramming confer them an advantage from normal cells^12–14^. Our studies among others contributed rehabiliting past studies from the german Nobel Prize winner, Otto Warburg, who introduced the idea of metabolic alteration in cancer cells^15^. In his seminal article, Warburg (1956) presented the concepts of “uncoupling of respiration” and “aerobic glycolysis” occurring in cancer cells, to explain that cancer cells rely on the cytoplasmic fermentation of glucose to lactic acid for energy (ATP) synthesis rather than the oxidative phosphorylation^16^. Otto Warburg explained this observation as a phenotypic expression of deleterious mitochondria. This characteristic of cancerous cells, labeled as the Warburg effect, has been the subject of extensive investigations the past years^17^. The Warburg effect confers a metabolic advantage to the cancerous tissue compared to normal somatic cells. This has been described as a consequence of the hybridic aspect of cancer cells metabolism where anabolism and catabolism occur simultaneously^14^. Carbon substrates are catabolized and intermediate compounds used as primary source for building blocks synthesis (lipids, proteins and nucleic acids), essential for cell growth^18–20^. This hybrid metabolism explains why targeting one pathway with a chemoterapeutic agent is barely enough to stop tumor growth. Indeed, metabolic and epigenetic reprogramming of cancer cells confer a metabolic plasticity in their central carbon metabolism (CCM), which could explain their acquired resistance to current chemotherapies.

To this end, an interesting approach would be to target the CCM using metabolic drugs known to inhibit specific enzymes. Among enzymes of the CCM, both pyruvate dehydrogenase (PDH) and ATP citrate lyase (ACL) play key role in metabolic reprogramming of cancer cells^18^. PDH enzymatic complex converts pyruvate to acetyl-CoA and fuels the tricarboxylic acid cycle in normal cells. In cancer cells, PDH has been shown to be inhibited whereas ACL is overexpressed. ATP citrate lyase converts cytoplasmic citrate to oxaloacetate and acetyl-CoA, a precursor for lipid synthesis. Other key enzymes playing pivotal role on cancer metabolism are lactate dehydrogenase (LDH) and enzymes involved on the electron transfer chain (ETC) reactions. Indeed, metabolic therapies targeting these enzymatic reactions involved in carbon resources fermentation could limit their uptake by the tumor in order to vanish the Warburg phenotype.

We used Alpha Lipoic Acid (ALA) and Hydroxycitrate (HCA), two old drugs from the pharmacopoeia targeting PDH and ACL, respectively. Interestingly, we managed to show that combination of ALA and HCA deeply inhibits cultures of three cancer cell lines (MBT-2 bladder carcinoma, B16-F10 melanoma and LL/2 lung carcinoma)^21^. These results agree with that from Hatzivassiliou and colleagues who reported the inhibition of cancer cells growth when using specific silencing RNA (SiRNA) to vanish ACL protein expression^22^. Similarly, a study from Bonnet and colleagues (2006) demonstrated the efficacy of a small molecule, Dichloroacetate, in restoring PDH activity in cancer cells^23^. Taken together, these experimental results show the potential of targeting enzymes involved in programming the Warburg effect. These therapeutic approaches show a similar efficacy as for conventional therapies but without any side effects. Furthermore, we have also investigated the efficacy of our drug combination, namely METABLOC (ALA and HCA), used in synergy with standard chemotherapy drugs such as Cisplatin or Methotrexane^24^. We reported an enhanced delay in tumor growth when Cisplatin and Methotrexane are applied in combination with METABLOC. A standardized screening method allow identifying the best drug combinations to asses their effects on cancer cells metabolism and tumor growth^25^. A pre-clinical investigation thus consisted to further evaluate the effect of METABLOC combined to new drugs (Metformin and Diclofenac) on the growth of transplanted LL/2 Lewis lung carcinoma into mice. The choice of these two drugs is based on their reported positive effect at slowing tumor growth. Metformin is an old drug commonly used in type II diabeties [Diabetes Prevention Program Research Group]. Emerging studies are showing the positive effect of using Metformin in cancer therapy^26–28^. Since Metformin is used as an hypoglycemic drug in type II diabeties, it slows down insulin secretion and cell proliferation by decoupling mitochondrial respiration throughout the ETC. Interestingly, retrospective epidemiological studies have shown that diabetic patients with long-term Metformin treatment have a reduced risk of developing cancer^26,29^. Moreover, another pre-clinical study showed an anti-proliferative effect of Metformin on tumor xenograft in mice^30^. Diclofenac is also an old drug currently used as an anti-inflammatory agent. Recent studies mentioned the potential use of Diclofenac in cancer treatment^31–33^. For instance, Gottfried *et al*. (2013) showed that Diclofenac impairs the Warburg effect by targeting glucose transport into cancer cells and inhibiting lactate dehydrogenase (LDH) and monocarboxylate Transporter 1 (MCT1). This results in decreased glucose uptake and lactate secretion.

In this work, we injected xenograft of LL/2 Lewis lung carcinoma cells into the peritoneal cavity of mice and we tested the effect of different combinations of drugs on the tumor volume evolution. Results show a decrease in LL/2 tumor volume when METABLOC is administrated in combination with Diclofenac and Metformin. The METABLOC effect is enhanced when Metformin is used at high-dose. Results are compared to the case of administration of a classic chemotherapeutic agent (Cisplatin), as a positive control. We also set up a kinetic metabolic model of tumor growth in order to characterize the effect of metabolic therapies on tumor metabolism. The model was not only able to simulate tumor growth in accordance with experimental data, but also allowed to simulate inhibition of growth after application of the metabolic therapy. This approach to kinetic modeling of tumor growth opens avenues to the identification of new metabolic targets.

## Results

### High-dose Metformin and Diclofenac slightly induce tumor regression

**Figure 1:**
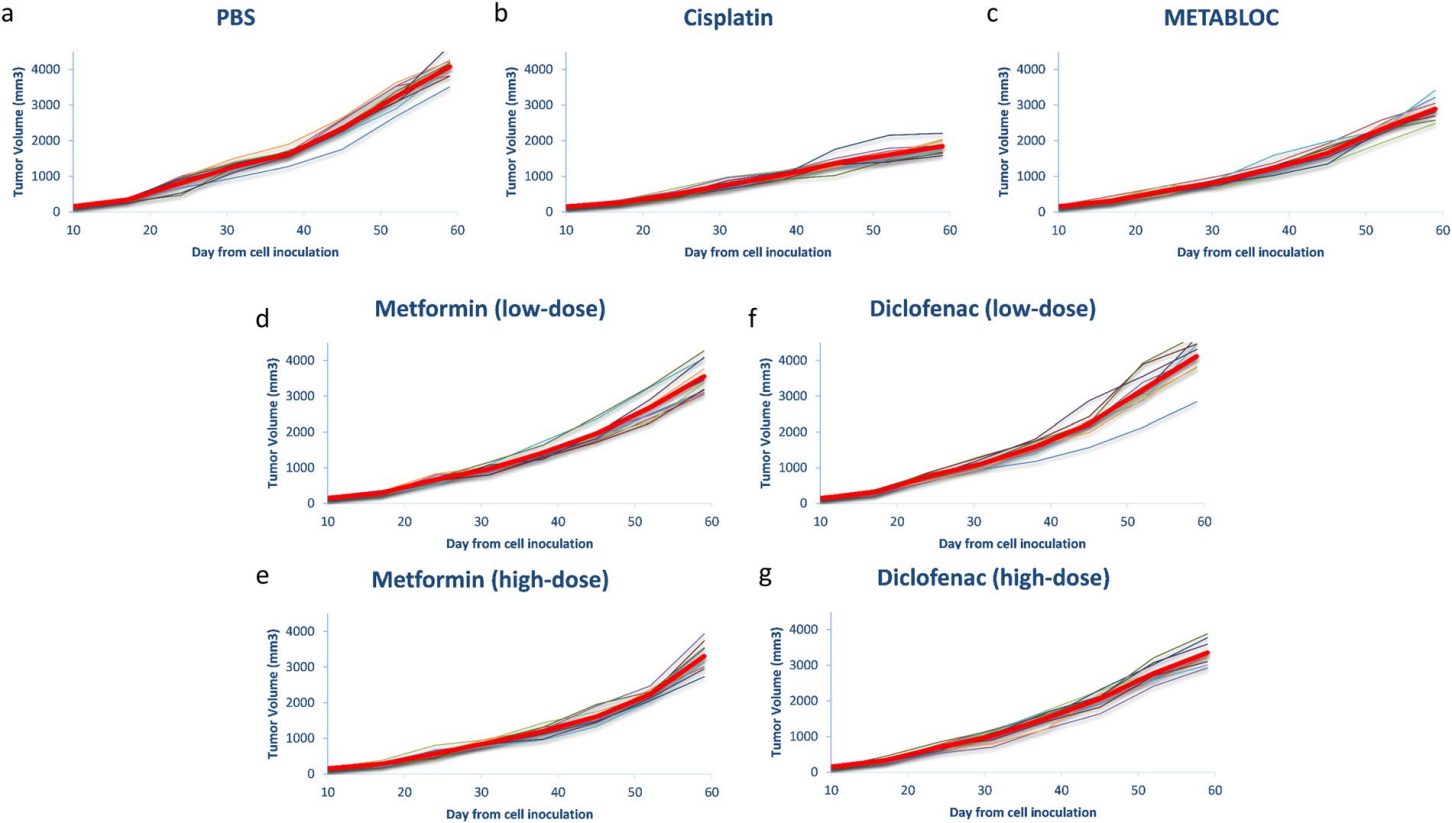
Xenograft tumor evolution in mice under metabolic therapies. Treatments started at day twelve after tumor inoculation in mice. The thin curves represent tumor volumes for each single mouse. The red bold curves are the mean values of all the thin curves. (**a**) Phosphate-buffered saline (PBS) is used as a control. (**b**) The chemotherapeutic agent, Cisplatin, slows down tumor growth. (**c**) Tumor growth is slightly reduced under METABLOC therapy. (**d**–**g**) Low-dose and high-dose Metformin and Diclofenac have no significant effects on tumor evolution.

We first analyzed the effect of exposing groups of C57BL/6 mice (n = 10) bearing the LL/2 Lewis lung tumor xenograft to chronic low and high-dose of Metformin and Diclofenac. As control, we also designed groups for chronic injection of Phosphate Buffer Saline (PBS), Cisplatin (chemoterapeutic drug) and METABLOC (calcium hydroxycitrate + lipoic acid). Results are reported in Fig. 1. Mice group treated with PBS, as negative control, reaches a tumor volume of 4000 mm^3^, whereas the group treated with Cisplatin (positive control) have half of the tumor volume at day 59 (Fig. 1a,b). We also applied chronic dose of METABLOC, already used in our previous studies. This combination of calcium citrate (HCA) and lipoic acid (ALA) does reduce tumor growth, as reported in our previous works (Fig. 1c). High-dose of Metformin and Diclofenac slightly delay tumor growth from day 10 to day 59 after cell inoculation (Fig. 1e,g). Low-dose metformin also delays tumor growth but not low-dose diclofenac (Fig. 1d,f). Metformin shows a dose-response effect on tumor volume (3500 mm^3^ for low-dose and 3300 mm^3^ for high-dose). This is not the case of Diclofenac-treated group where the high-dose Diclofenac slightly reduces the tumor volume (mean value = 3300 mm^3^) but the low-dose group is similar to PBS at day 59 (Fig. 1). Experiments were stopped at day 59 since PBS group reaches a lethal tumor volume. Then another group were fed with METABLOC and Metformin or Diclofenac.

### METABLOC and high-dose Metformin combination slows and inhibits tumor growth

**Figure 2:**
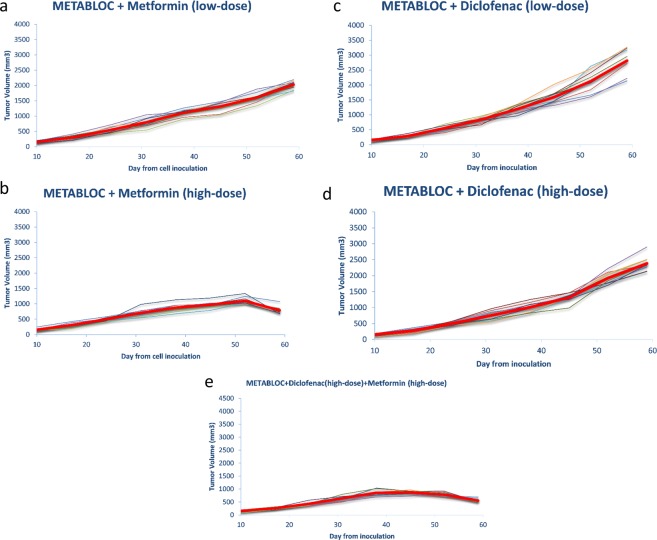
Tumor evolution under metabolic therapy combinations. The thin curves represent tumor volumes for each single mouse. The red bold curves are the mean values of all the thin curves. (**a**) METABLOC + low-dose Metformin slows down tumor growth. (**b**) METABLOC + high-dose Metformin significantly reduces tumor evolution and inhibits tumor growth at day 50 from cell inoculation. (**c**,**d**) METABLOC + low-dose and high-dose Diclonenac has no significant effect on tumor growth inhibition. (**e**) METABLOC + high-dose Metformin and Diclofenac drastically inhibits tumor growth and reverses the tumor curve.

METABLOC combination with high-dose Metformin and Diclofenac significantly delays LL/2 carcinoma progression in mice compared to PBS or METABLOC treatment (Fig. 2b,c). METABLOC with low-dose Metformin also have a positive effect whereas its combination with low-dose diclofenac has poor effect (Fig. 2d). Among these curves, only METABLOC and high-dose Metformin treated group shows a regression in tumor volume (Fig. 2b). The mean tumor volume is significantly decreased from day 45 to day 59 in the case of METABLOC + Metformin (high-dose) combination (p < 0.005). The tumor has a better response to our combination compared to Cisplatin treatment (p < 0.001). High-dose Metformin clearly enhances our previous METABLOC combination (Hydroxycitrate + Lipoic acid).

### High-dose Diclofenac enhances Metformin effect in inhibiting tumor growth

We went further in our investigation and treated a group of mice (n = 10) with full combination of METABLOC + Metformin (low and high-dose) + Diclofenac. Addition of high-dose Diclofenac to METABLOC + high-dose Metformin improves the tumor response to treatment (Fig. 2e). Tumor volume starts regressing at day 45 in METABLOCS (METABLOC + Metformin (high-dose) + Diclofenac (high-dose)) treated group whereas that regression is observed at day 52 for METABLOC + Metformin (high-dose) (Fig. 2b). Combination with low-dose Metformin is less pronounced. METABLOCS significantly decreases tumor growth at day 59 compared to METABLOC + high-dose Metformin (p < 0.001) (Fig. 2b). Compared to Cisplatin-treatment, METABLOC has less effect but tumor cell sensitivity increases when the last is combined with metformin and then with Metformin + Diclofenac. Combinations of these old and low-cost drugs are two-times more efficient than the classic chemotherapeutic agent. Our new finding is that a combination of METABLOC (Hydroxycitrate + Lipoic acid) and high-dose Metformin strongly inhibits growth of tumor xenograft inoculated to a group of mice (n = 10).

### *In silico* metabolic therapy simulates tumor growth inhibition

**Figure 3:**
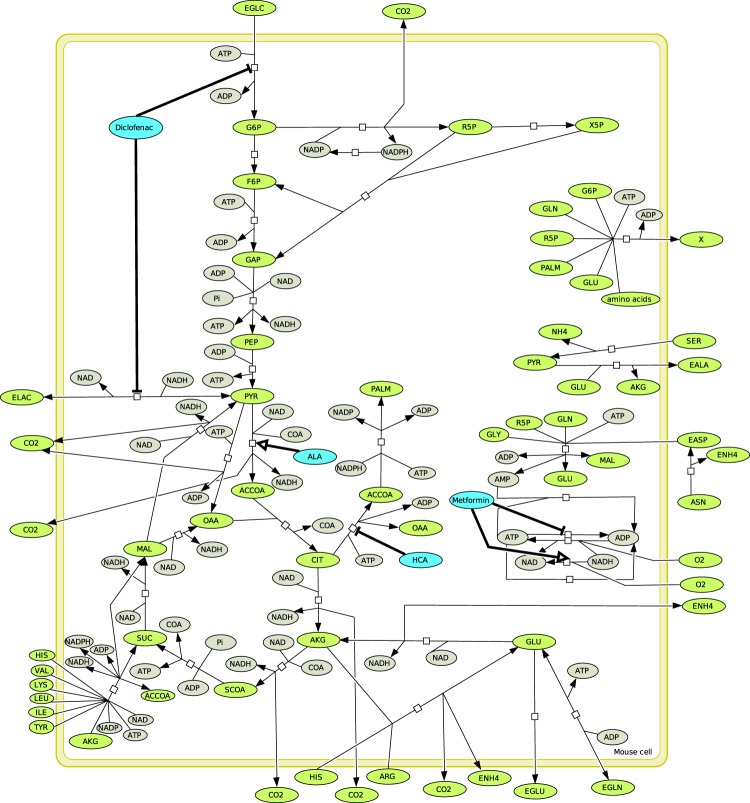
The metabolic network. The core metabolism of cancer cells integrates glycolysis, the pentose phosphate pathway, the citric acid cycle, lipogenesis and amino acids metabolism. Biomass synthesis is described as the incorporation of amino acids, intermediate metabolites and palmitic acid. The metabolic therapy includes Diclofenac as hexokinase and lactate dehydrogenase inhibitor; alpha-Lipoic acid (ALA) as pyruvate dehydrogenase activator; Hydroxycitrate (HCA) as ATP citrate lyase inhibitor; Metformin as ATP synthase inhibitor and NAD leakage activator. Notations: ACCOA: Acetyl-CoenzymeA, ACCOA: Acetyl-CoenzymeA, ACL: ATP-citrate lyase, ADP: Adenosine diphosphate, AK: Adenylate kinase, AKG: *α*-ketoglutarate, ALA: Extracellular alanine, AMP: Adenosine monophosphate, ARG: Extracellular arginine, ASN: Asparaginase, ASP: Extracellular aspartate, ATP: Adenosine triphosphate, CIT: Citrate, CO_2_: Carbone dioxide, DICLO: Extracellular diclofenac, EGLC: Extracellular glucose, EGLN: Extracellular glutamine, EGLU: Extracellular glutamate, F6P: Fructose-6- Phosphate, FADH2: Flavine adenine dinucleotide, FUM: fumarate, G6P: Glucose-6-phosphate, GAP: Glyceraldehyde 3-phosphate, concentration, GlnT: Glutamine synthetase, GLU: Glutamate, GLY: Extracellular Glycine, H_2_O: Hydrogen dioxide, HCIT: Extracellular hydroxicitrate, HIS: Extracellular histidine, HISARGTA: Histidine/arginine transamination, ILE: Extracellular isoleucine, LA: Extracellular lipoic acid, LAC: Extracellular lactate, LEU: Extracellular leucine, LYS: Extracellular lysine, MAL: Malate, ME: Malic enzyme, Metformin: Extracellular metformin, NAD: Nicotinamide adenine dinucleotide (Oxidized), NADH: Nicotinamide adenine dinucleotide (reduced), NADP: nictoniamide adenine dinucleottide phosphate, NADPH: nictoniamide adenine dinucleotide phosphate (reduced), NH_4_: Extracellular ammonia, OXA: Oxaloacetate, PALM: Palmitate, PEP: Phosphoenolpyruvate, Pi: inorganic phosphate, PK: Pyruvate kinase, PPRibP: Nucleotide synthesis, PYR: Pyruvate, R5P: Ribulose-5-phosphate, SER: Extracellular serine, SUC: succinate, SUCCOA: Succinyl coenzyme A, THR: Extracellular threonine, TYR: Extracellular tyrosine, VAL: Extracellular valine.

A kinetic metabolic model (see the metabolic network in Fig. 3 and the system of differential equations in methods) has been set up from previous models describing the simulation of Chinese Hamster Ovary cells (CHO) growth on different media for the production of monoclonal antibodies^34^. Here, our model is first used to simulate the growth of a tumor xenograft implanted to a group of mice. Then, the model was used to simulate the impact of *in silico* metabolic therapy - METABLOC - Metformin - Diclofenac on tumor growth. This model is not only useful for simulating experimentally obtained tumor growth, but it also allowed us to characterize the metabolic phenotypes the tumor developing in mice with time.

**Figure 4:**
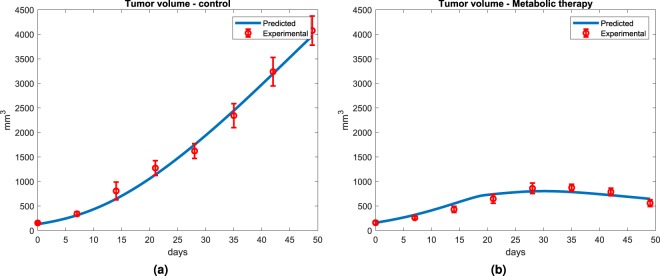
Predictions of the tumor growth in control and under*in silico* metabolic therapy. (**a**) The kinetic model of tumor metabolism simulates tumor volume in line with the experimental data. (**b**) Under metabolic therapies (METABLOC combined with diclofenac and high dose of Metformin), the model predicts tumor inhibition and its volume regression, as determined experimentally.

The model simulates adequately tumor xenograft growth in mice for the most efficient treatment strategy (Fig. 4a). The predicted tumor volume is within the standard deviations of experimental tumor volume. The model predicts a tumor volume evolution from 154 mm^3^ to 3900 mm^3^, in line with the experimental data (154 ± 16 to 4076 ± 297 mm^3^). It is important to note that model predictions are only based on total tumor cell volume and blood network of capillaries. Results thus confirming model ability to describe tumor growth. For the model integrating metabolic therapy, the therapeutic molecules are supplied within the blood inlet flux (F) feeding the capillaries blood volume (i.e. tumor cells microenvironment). These molecules are known to specifically target specific enzymatic pathways; with the known effects on cell metabolism that are explicitly described in the model. Model simulations describe the inhibition of tumor growth by metabolic therapy (METABLOC + Metformin + Diclofenac) Fig. 4b. Both experimental and predicted data of tumor evolution show a reduced growth phase from t0 (10 days after tumor inoculation) and reach a plateau at day 35. Model simulations show a growth plateau at around 873 mm^3^ as for the experimental data (873 ± 70 mm^3^). However, the model does not simulate the decrease in tumor volume. This is probably due to the occurence of apoptosis in the tumor, a phenomenon that is not described in the model.

### The metabolic model predicts reversed Warburg effect upon metabolic therapy

**Figure 5:**
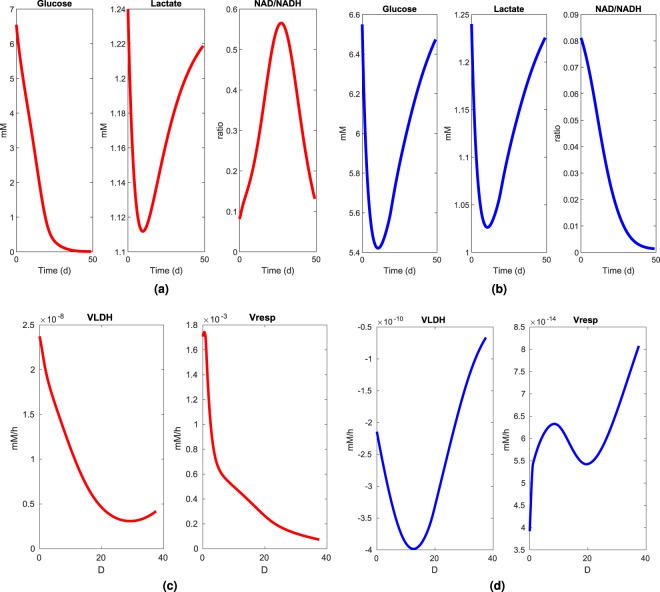
Simulated blood metabolites (glucose and lactate) and simulated redox ratio (NAD^+^/NADH) in tumor (**a**) and tumor treated with metabolic drugs (**b**). Simulated fluxes of lactate dehydrogenase (VLDH) and respiration (Vresp) in tumor (**c**) and tumor treated with metabolic drugs (**d**). (**a**) Lactic acid is first eliminated by the blood flow and then accumulates with tumor growth. NAD^+^/NADH ratio decreases upon glucose exhaustion. (**b**) Under metabolic therapy glucose uptake slows down, as well as lactic acid secretion. NAD^+^/NADH ratio decreases with tumor growth. (**c**) The high value of VLDH and the decrease of the respiration flux are markers of the Warburg effect in the tumor. (**d**) The negative flux of VLDH which corresponds to lactate uptake and the increase of the respiration flux both translate a reverse Warburg effect.

In a recent work^35^, we reported concentrations of key metabolites involved in energy management throughout the central carbon metabolism. Concentrations of adenosine triphosphate (ATP), nicotinamide adenine dinucleotide (NAD), and nicotinamide adenine dinucleotide phosphate (NADP) have been measured in normal and cancer cells, extracted from fresh human colon tissues. Our results showed high NAD^+^-to-NADH and NADP^+^-to-NADPH redox ratios in cancer cell populations compared to normal proliferating cells. Interestingly enough, our model also predicts the high throughput aerobic glycolysis, commonly known as the Warburg effect, observed in cancer cells (Fig. 5(a,c)). Consumption of glucose by the tumor cells is linear, until reaching limitation level at the end of the 25th day and totally consumed 59 days after incubation. This accelerated glycolysis results in a massive release and accumulation of lactate in the blood stream. NAD^+^-to-NADH is the catabolic marker of that aerobic glycolysis proposed by Warburg in his seminal article^16^. Therefore, an important NAD^+^-to-NADH ratio allows the maintenance of the glycolytic potential of cancer cells. Combining METABLOC and high-dose of Metformin and Diclonenac considerably reduces glucose consumption by tumor cells. This phenomenon thus results in decreased lactate secretion and accumulation (Fig. 5(b,d)). NAD^+^-to-NADH redox ratio is low and decreases with metabolic therapies. These observations are both signatures of a reduced glycolytic flux or the reverse Warburg effect.

## Conclusion

Metabolic therapies have been used to study tumor xenograph growth in mice. We experimentally showed that the combination of METABLOC, high-dose Metformin and Diclofenac inhibits the tumor growth. We proposed a dynamic metabolic model of the tumor which simulates the effects of the metabolic therapies on tumor evolution and tumor cell metabolism. This model also predicts tumor regression, a lower glycolytic flux and lactic acid secretion upon metabolic treatments. Intracellular NAD^+^/NADH redox ratio is low and decreases, as a consequence of reduced glycolytic flux. Indeed, the oxidative phosphorylation may be rewired from the fermentation pathway, favoring mitochondrial respiration and inversed Warburg effect. Our model supports our experimental results and it can also be use to test new combination of metabolic treatments. These results pave the way for new strategies in metabolic therapy and *in silico* metabolic drug design.

## Methods

### Cell culture

The LLC/1 cell-line ATCC® CRL-1642 (LGC Standards, Molsheim-France) cultivated in the medium DMEM Glutamax I (Invitrogen-Gibco) with 10% of FBS (Eurobio) and 1/10000 IU of Penicillin/Streptomycin (Sigma Aldrich P4333) were injected into the peritoneal cavity of mice at 5 million cells per mouse in a 150 shot and within 12 days of incubation, when tumors reached 130 mm^3^, mice were randomized and the treatments were started.

### Metabolic treatment

Lipoic acid (Sigma Aldrich T1395), calcium hydroxycitrate(IWDO) and cisplatin (Sigma Aldrich 479306) were dissolved in PBS and injected intraperitoneally (i.p.) two times per day (2X/D) for lipoic acid and calcium hydroxycitrate, 1X/2D for cisplatin at 10 mg/kg, 250 mg/kg and 1 mg/kg, respectively. Drops of ethanol were added to completely dissolve lipoic acid in PBS solution. Diclofenac sodium (Sigma-Aldrich 93484) at 3 mg/kg and 30 mg/kg adjusted in 25 *μ*L/mL (LOW) and 250 *μ*L/mL (HIGH) of drinking water were administered orally (Per Os). A mouse drinks about 3 mL water per day. Metformin (Sigma-Aldrich 1396309) at 2.5 mg/kg, 12.5 mg/kg and 25 mg/kg adjusted in 20 *μ*/mL (LOW), 100 *μ*L/mL (MEDIUM) and 200 *μ*L/mL (HIGH) of drinking water were also administered orally.

### Animal handling

Mice were divided in a weight-normalized manner into the groups of 10 animals per group. Forty eight groups of 6–8 weeks old inbred male C57BL/6 mice (mean weight: 21.15 ± 1.11 g) were obtained from Pasteur Institute, Iran. They were housed five to a cage with access to autoclaved mouse chow and water *ad libitum*. They were kept in a room under controlled temperature (22 °C), humidity (55%) and light (lights on 7 h00 am–7 h00 pm). All animals received human care in compliance with the Guide for the Care and Use of Laboratory Animals [DHEW Publication No. (NIH) 85–23, from 1985 with all next updates, Office of Science and Health Reports, DRR/NIH, Bethesda, MD 20205]. The experimental protocols were approved by the company Nosco Pharmaceuticals.

### The metabolic network

The metabolic network presented here and displayed in Fig. 3 has been modified from previous models of Chinese Hamster Ovary cells (CHO) and mouse myeloid derived suppressor cells (MD-SCs)^34,36–39^. It includes 35 enzymatic reactions describing the fate of 52 metabolites. We distinguished two compartments: external (i.e. blood microenvironment of tumor cells) and intracellular cell metabolites, with the cell as a unique compartment. The network integrates pathways of the central carbon metabolism (CCM) such as glycolysis, the pentose phosphate pathway, the tricarboxylic acid cycle, lipogenesis, the oxidative phosphorylation and pathways of amino acid metabolism. In this CCM, glucose and amino acids are the main sources of carbon and nitrogen for cell proliferation and biomass synthesis. For further details see^34^.

### Minimal cut sets of the metabolic networks

The concept of minimal cut set has been introduced to determine the minimal set of reactions whose deletion completely blocks a target^40^. In our metabolic network, there are 1058 minimal cut sets which prevent the tumor growth (X). They have been calculated with CellNetAnalyzer^41,42^ and we have selected the shortest. Twenty of them have their size lower than two and only 6 are of size one: VHK, VG6PDH, VCS, VACL, VPALM, Vgrowth. As our therapy is not a genetic modification, the inhibition of one of them will not totally prevent the tumor growth. This structural analysis shows that the diclofenac which inhibits the hexokinase (VHK), and the hydroxycitrate which inhibits the ATP citrate lyase (VACL), should have a negative impact on the tumor growth.

### Dynamic modeling of tumor volume

**Figure 6:**
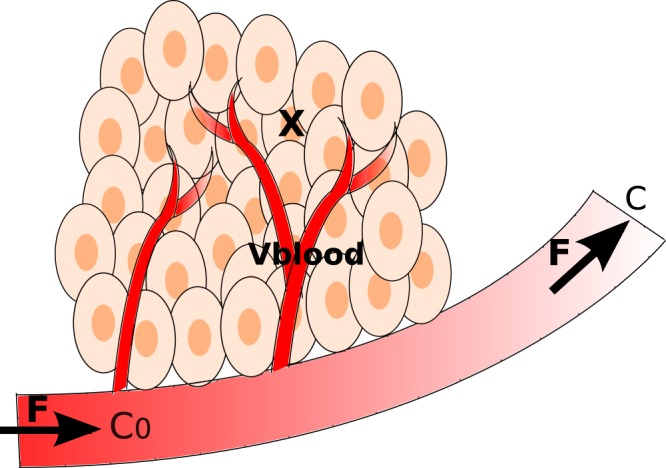
Tumor microenvironment assimilated to a perfusion reactor.

The tumor volume includes cancer cells and the network of capillaries (Fig. 6). The model was established on the basis of mass balances on metabolites and tumor cells concentration. The tumor cells are continuously perfused from a network of capillary of total volume *V*_*b*_, which is considered as a perfectly mixed stirred-tank reactor (CSTR). The blood composition within the tumor volume was thus considered homogeneous. In a perfusion system the cells are retained within the vessel.

The tumor is continuously fed from the network of capillaries, with a global blood flow rate (*F*) of composition *C*_0_ at tumor inlet. For simplification purposes, the variation of the blood flow with tumor growth was neglected. Here, *C* and *C*_0_ are the vectors of tumor blood concentrations and blood concentrations in metabolites at tumor inlet, respectively (in mM), and *m* is the vector of concentrations of tumor cells intracellular metabolites (in mM). *S*_*c*_ and *S*_*m*_ are the stoichiometric matrices of the reactions involving the extracellular and intracellular metabolites, respectively. The *r* term is the specific rates vector (in mM/h in cells and mmol/10^6^ cells/h in capillaries) of each reaction in the metabolic network, whilst *μ* and *μ*_*blood*_ are the cells and capillaries specific growth or volume increase rates (*h*^−1^), respectively. *X* is the total tumor cells volume (in 10^6^ cells/ml) and *ε*_*m*_ is the fraction of intracellular metabolites that are integrated into the cells matter. The dynamical system is written as follow and is explained in supplementary materials:

$$\{\begin{array}{lcl}\frac{dC}{dt} & = & {S}_{c}\times r\times \frac{X}{{V}_{blood}}+\frac{({C}_{0}-C)\times F}{Vblood}-{\mu }_{blood}\times C\\ \frac{dm}{dt} & = & {S}_{m}\times r-({\varepsilon }_{m}+m)\times \mu \\ \frac{dX}{dt} & = & \mu \times X-{k}_{d}\times \frac{1-{(\frac{NAD}{NADH})}^{n}}{{({k}_{\frac{NAD}{NADH}})}^{n}+{(\frac{NAD}{NADH})}^{n}}\times X\\ \frac{dVblood}{dt} & = & {\mu }_{blood}.{V}_{blood}\\ \frac{d{V}_{tumor}}{dt} & = & \frac{dX}{dt}+\frac{dVblood}{dt}\end{array}$$

### Parameter estimation

Parameters value where first taken from previous works on CHO cells^34,36^, when not available for cancer or human cells in the literature or databanks. Then, we used nonlinear optimization function, *fmincon* (MatWorks®), to optimize sensitive parameters which are *F*_*in*_, *F*_*out*_ and the specific growth rate of tumor cells (*μ*). Parameters value optimization was based on the following objective function:

$$\min (\sum _{n=1}^{N}\,\sum _{t=1}^{T}\,{(\frac{{Y}_{n,t}^{\exp }-{Y}_{n,t}(p)}{{\sigma }_{n,t}})}^{2})$$

where ${Y}_{n,t}^{exp}$ is the *n*^*th*^ experimental data at the *t*^*th*^ experimental time, *Y*_*n,t*_(*p*) the simulated output with “p” a vector of the three sensitive parameters and “*σ*” the standard deviation of the experimental measurements.

## Supplementary information

Dynamical system description

Dataset 1
