# Supplementary material for: Metabolic therapies inhibit tumor growth in vivo and in silico
Source: Sci Rep. 2019 Feb 28;9:3153. doi: 10.1038/s41598-019-39109-1 (PMC6395653; doi:10.1038/s41598-019-39109-1)
Supplement: Supplementary file 1 — Dynamical system description [file 41598_2019_39109_MOESM1_ESM.pdf]

Supplementary Information

Metabolic therapies inhibit tumor growth *in vivo* and *in silico*

Jorgelindo da Veiga Moreira[1*], Minoo Hamraz[4], Mohammad Abolhassani[5]
Laurent Schwartz[6], Mario Jolicoeur[1], Sabine Peres[2,3]

1 Research Laboratory in Applied Metabolic Engineering, Department of Chemical Engineering, École Polytechnique de Montréal, P.O. Box 6079, Centre-ville Station, Montreal (Quebec), Canada
2 LRI, Université Paris-Sud, CNRS, Université Paris-Saclay, 91405 Orsay, France
3 MaIAGE, INRA, Université Paris-Saclay, 78350 Jouy-en-Josas, France
4 Institut Cochin, Université Paris- Descartes, 75014 Paris, France
5 Nosco Pharmaceuticals, 75015 Paris, France
6 Assistance Publique des Hôpitaux de Paris, 149 avenue Victoria 75004 Paris, France

Dynamical system description

The metabolite concentrations evolution were developed from following the nomenclature proposed in[1]:
Rate of accumulation = Rate of inflow - Rate of outflow + Rate of generation - Rate of consumption

Extracellular metabolites (C) balance equation: A mass balance was performed on every extracellular metabolites described in the network. The extracellular metabolites concentration are in mM.

$\frac{d(V_{blood} \times C)}{dt} = F \times (C_0 - C) + S_r \times r \times X$
By developing, we thus obtain:

$$\frac{dC}{dt} = S_c.r.\frac{X}{V_{blood}} + \frac{(C_0 - C).F}{Vblood} - \mu_{blood}.C$$

with $\mu_{blood} = \frac{1}{V_{blood}} \frac{dV_{blood}}{dt}$

Intracellular metabolites (m) balance equation A mass balance was performed on every intracellular metabolites described in the network. The intracellular metabolites concentration are in mmol/10^6 tumor cells.

$\frac{dmX}{dt} = S_m \times r \times X - \epsilon_m \times \mu \times X$
with ϵ_m: biomass integration coefficient, , which accounts for the intracellular metabolites that are consumed for the synthesis of the cell macromolecules such as DNA, proteins and lipid.
By developing, we thus obtain:

$$\frac{dm}{dt} = S_m \times r - (\epsilon_m + m) \times \mu$$

Tumor cells (X) balance equation A mass balance was performed on tumor cells, considering that the tumor mass is continuously feed by a blood flow, which carries all nutrients, at the inlet, and the waste metabolites, at the outlet. We consider a death rate k_d which is assimilated with an allosteric effect on the ratio $\frac{NAD}{NADH}$.

We thus obtain :

$$\frac{dX}{dt} = \mu \times X - k_d \times \frac{1 - (\frac{NAD}{NADH})^n}{(k_{\frac{NAD}{NADH}})^n + (\frac{NAD}{NADH})^n} \times X$$

References

[1] Bailey, J. & Ollis, D. Biochemical engineering fundamentals. *Second Edition. McGraw-Hill* (1986).
