# Supplementary figures and images for: Metabolic therapies inhibit tumor growth in vivo and in silico
Source: Sci Rep. 2019 Feb 28;9:3153. doi: 10.1038/s41598-019-39109-1 (PMC6395653; doi:10.1038/s41598-019-39109-1)

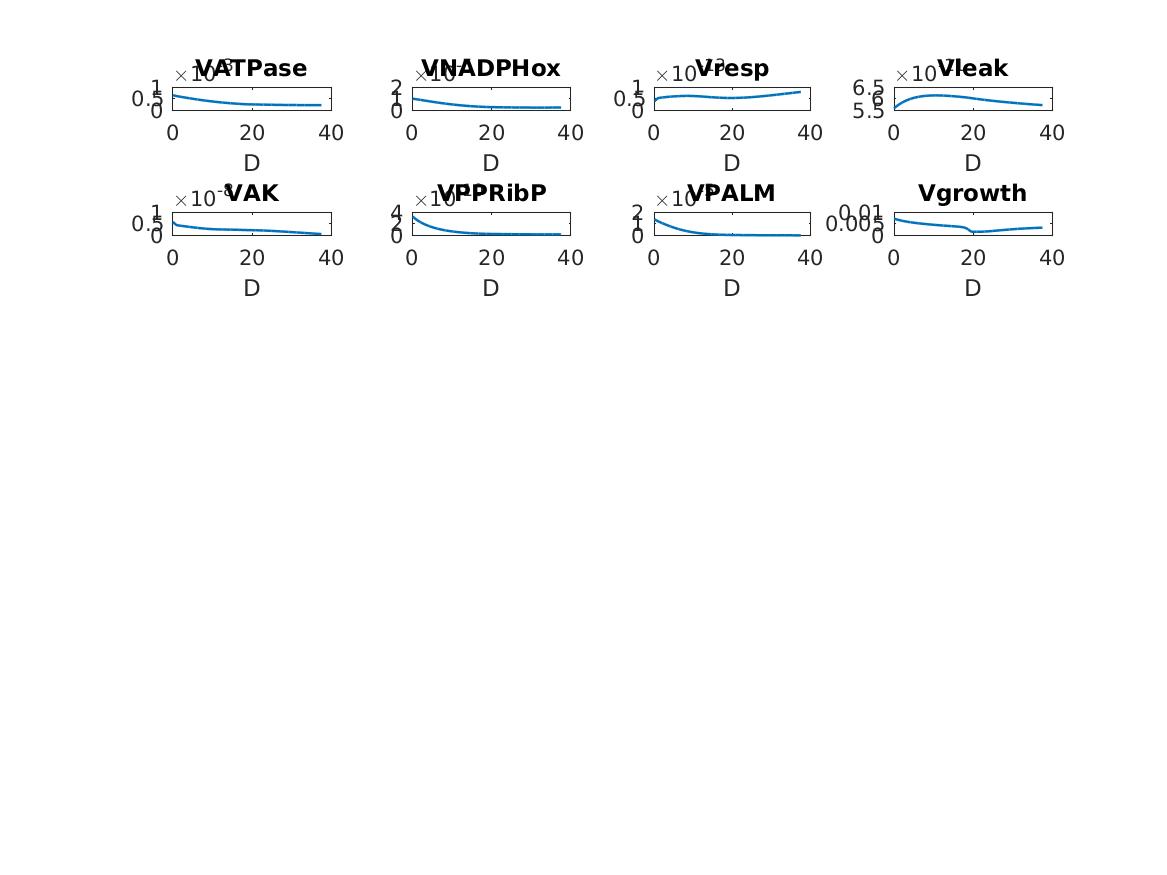

Supplement: Supplementary file 2 — Dataset 1 [file 41598_2019_39109_MOESM2_ESM.zip › MetabolicTherapiesModel/Figures/Figures_Flux_Drugs/figure_4.jpg]

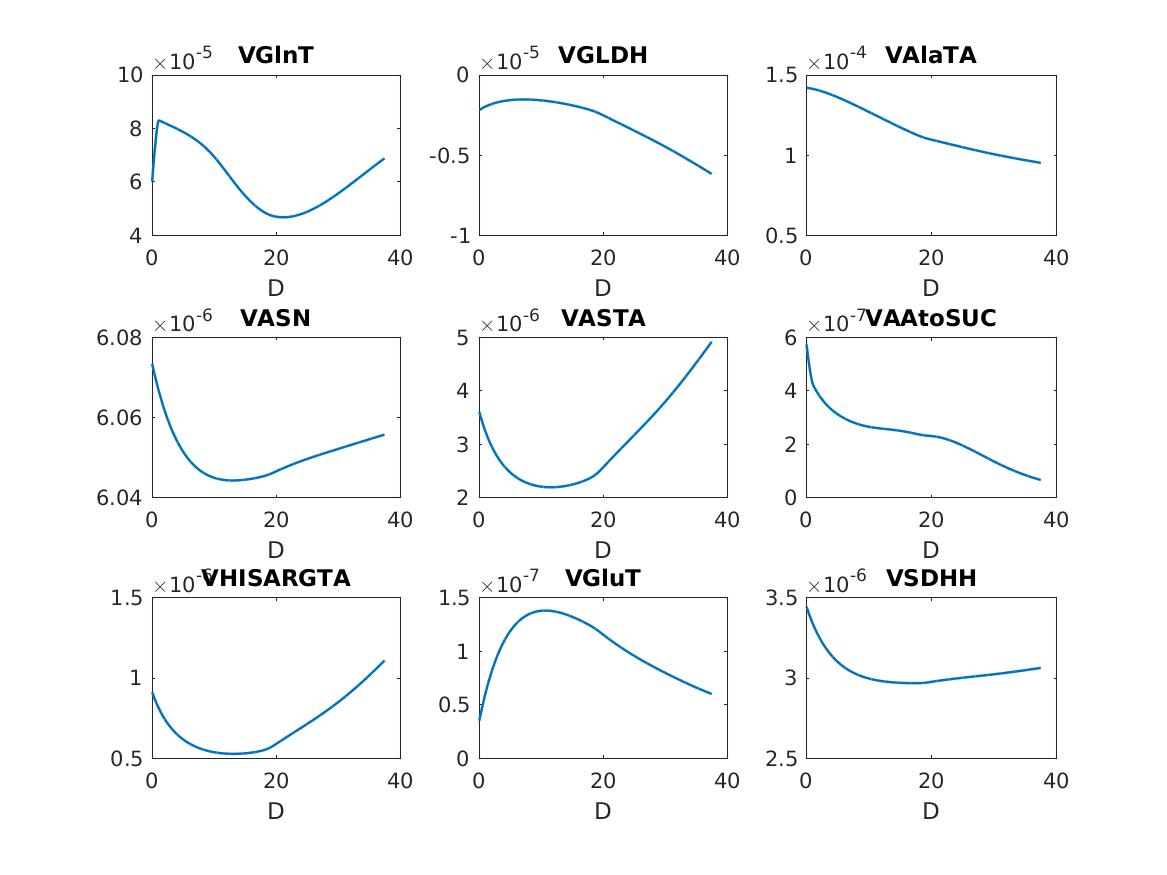

Supplement: Supplementary file 2 — Dataset 1 [file 41598_2019_39109_MOESM2_ESM.zip › MetabolicTherapiesModel/Figures/Figures_Flux_Drugs/figure_3.jpg]

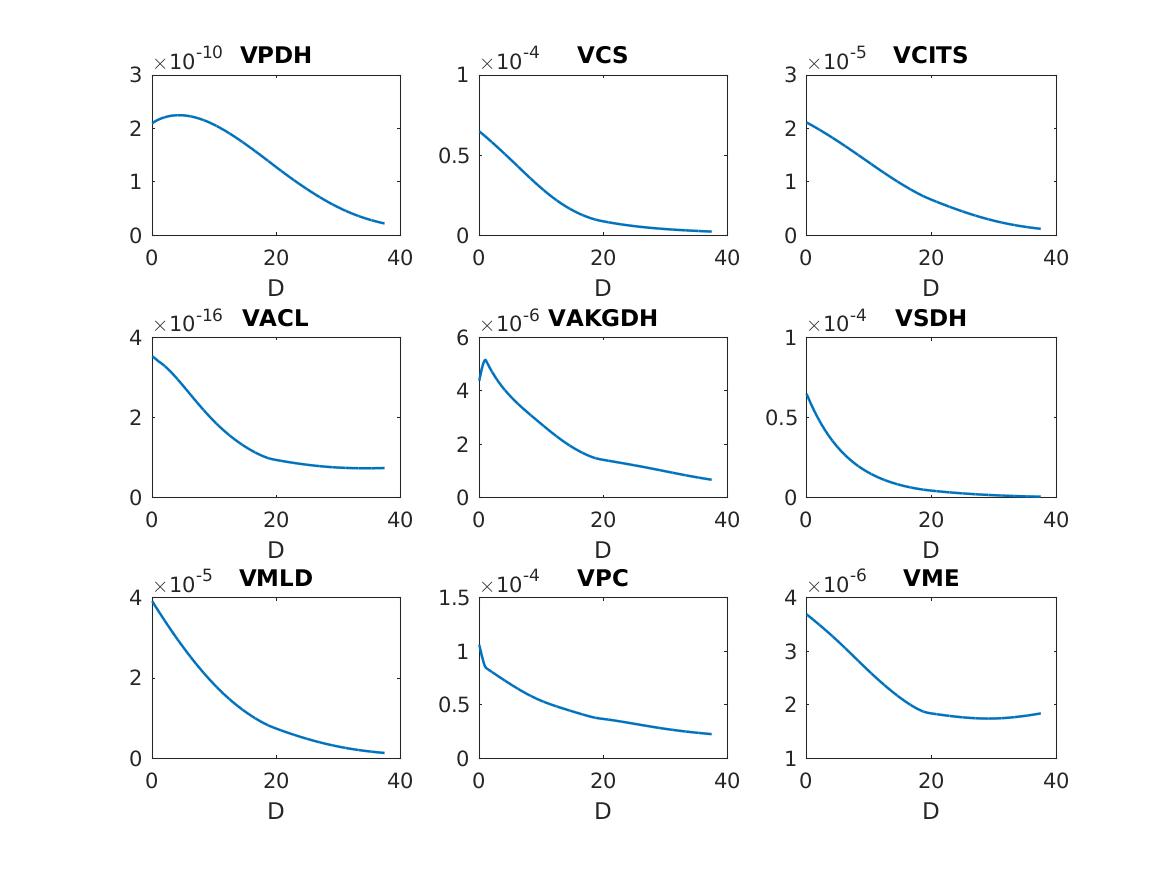

Supplement: Supplementary file 2 — Dataset 1 [file 41598_2019_39109_MOESM2_ESM.zip › MetabolicTherapiesModel/Figures/Figures_Flux_Drugs/figure_2.jpg]

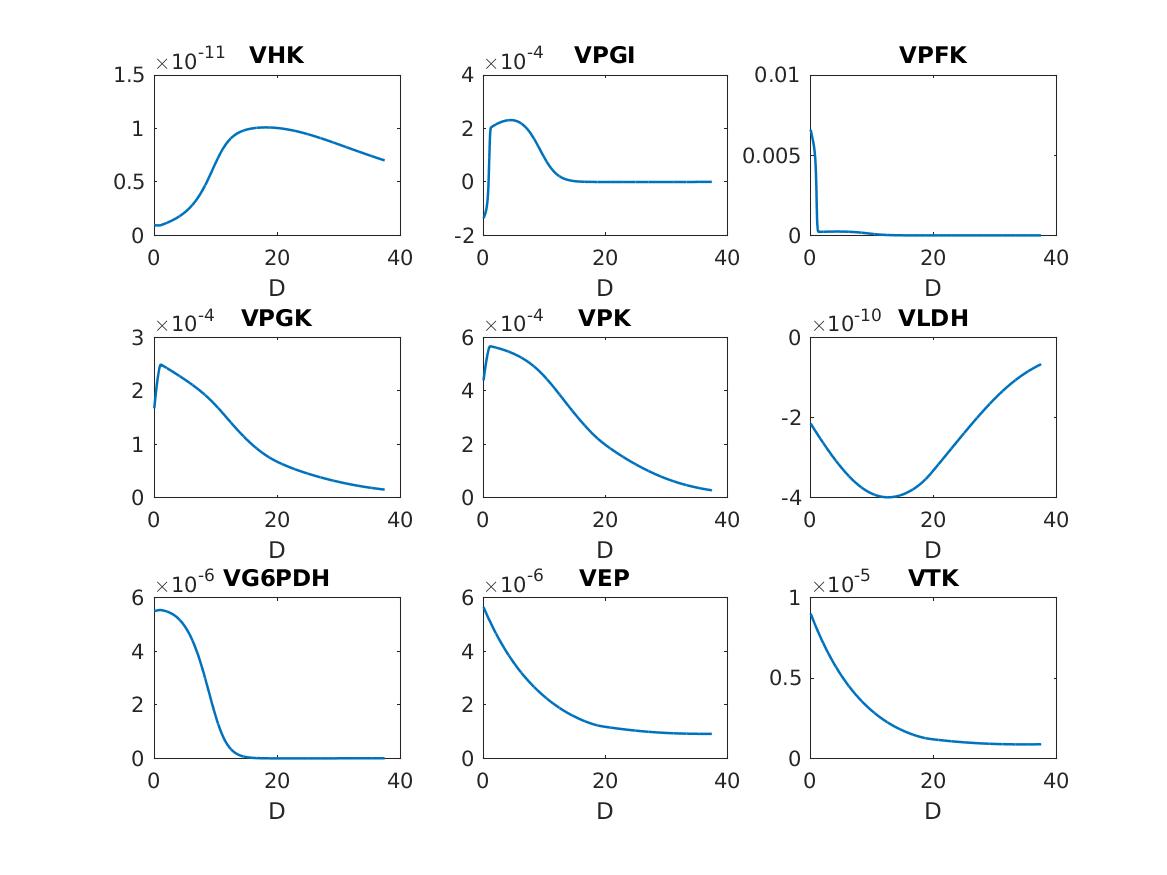

Supplement: Supplementary file 2 — Dataset 1 [file 41598_2019_39109_MOESM2_ESM.zip › MetabolicTherapiesModel/Figures/Figures_Flux_Drugs/figure_1.jpg]

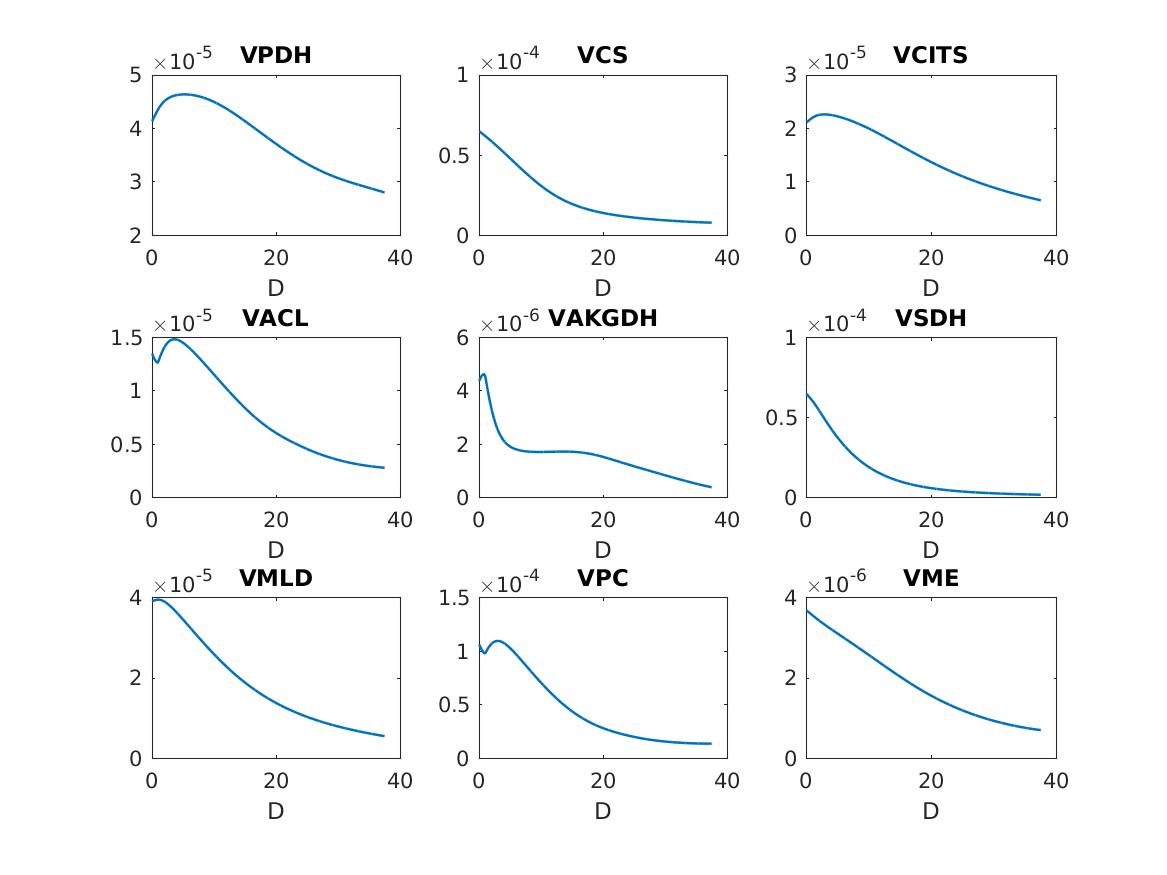

Supplement: Supplementary file 2 — Dataset 1 [file 41598_2019_39109_MOESM2_ESM.zip › MetabolicTherapiesModel/Figures/Figures_Flux_noDrugs/figure_2.jpg]

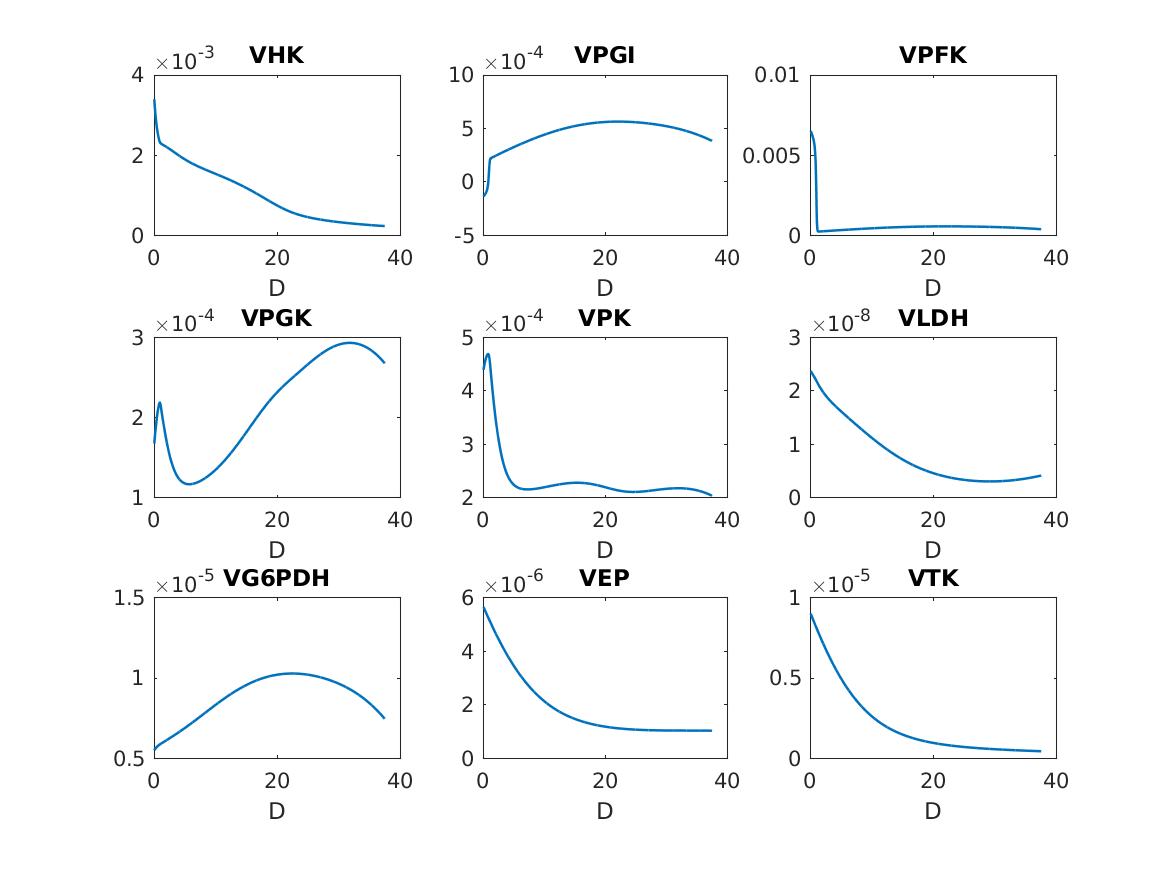

Supplement: Supplementary file 2 — Dataset 1 [file 41598_2019_39109_MOESM2_ESM.zip › MetabolicTherapiesModel/Figures/Figures_Flux_noDrugs/figure_1.jpg]

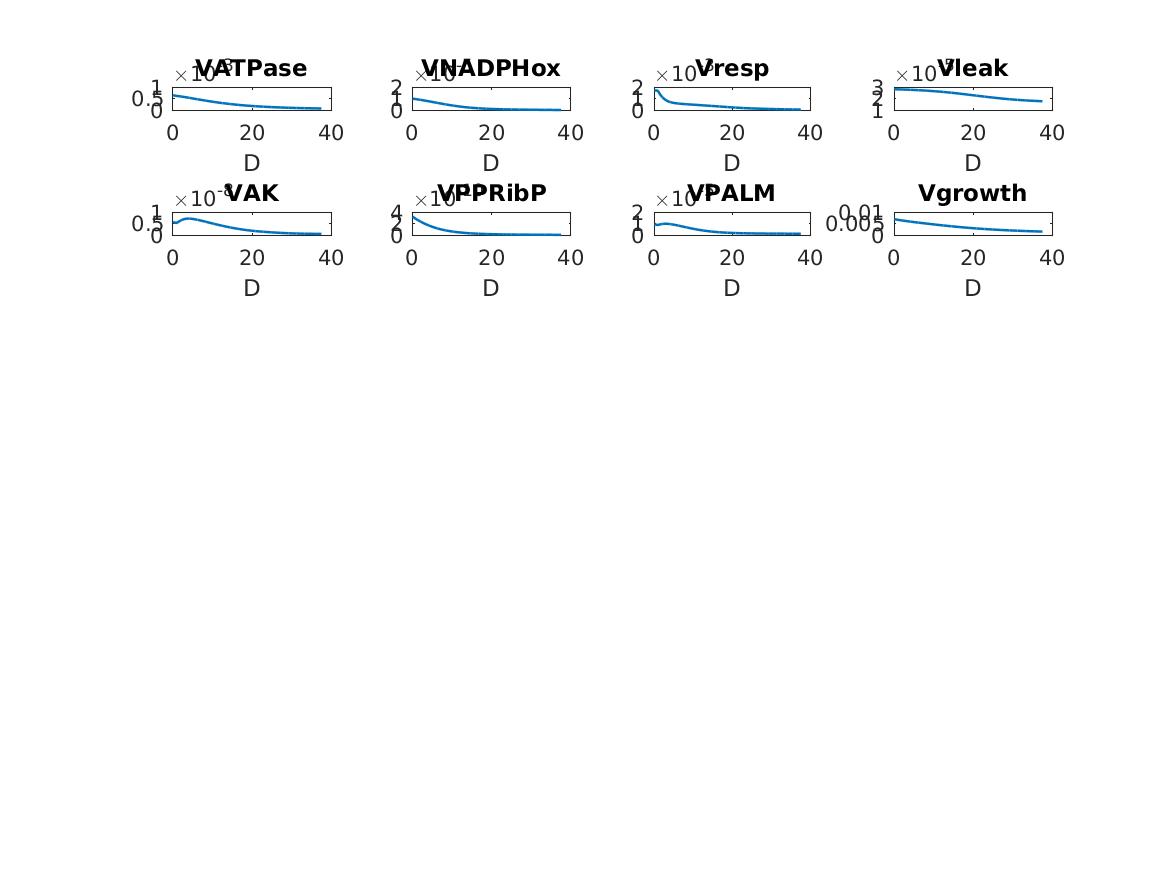

Supplement: Supplementary file 2 — Dataset 1 [file 41598_2019_39109_MOESM2_ESM.zip › MetabolicTherapiesModel/Figures/Figures_Flux_noDrugs/figure_4.jpg]

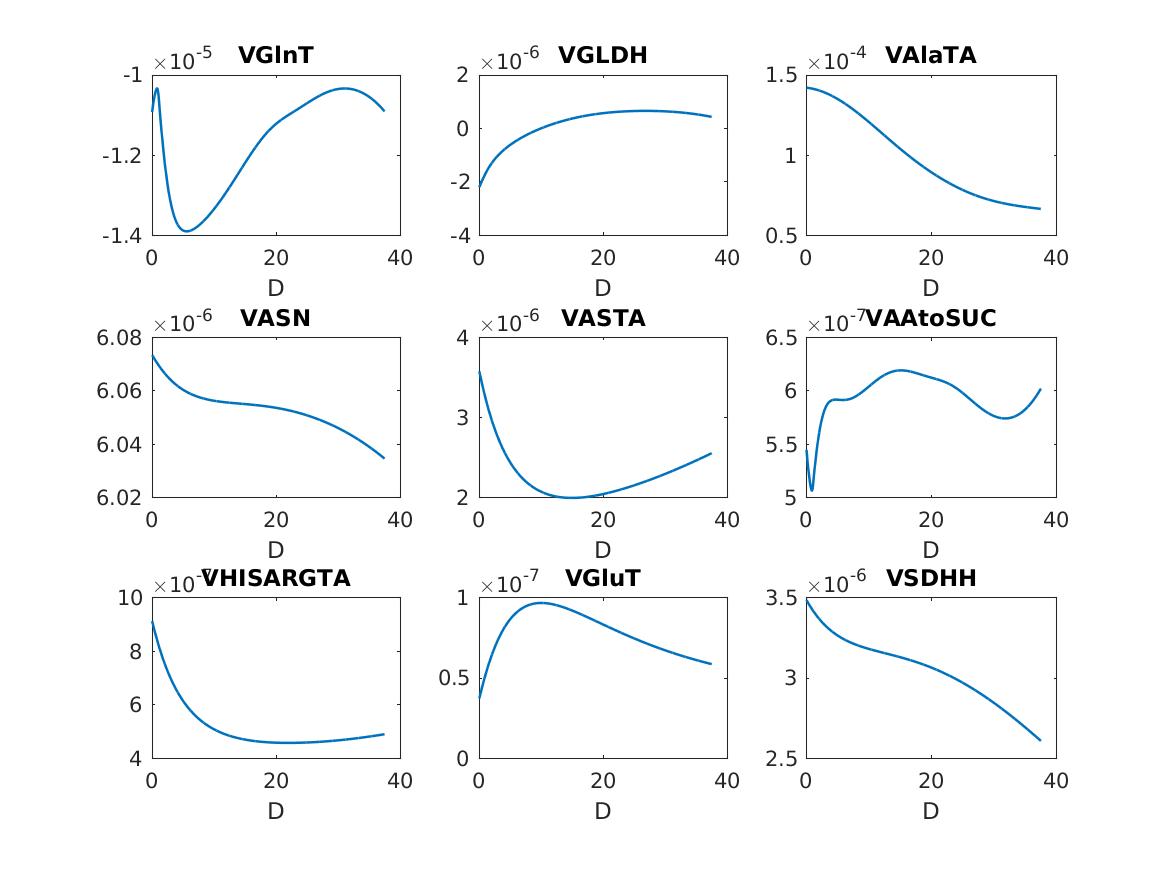

Supplement: Supplementary file 2 — Dataset 1 [file 41598_2019_39109_MOESM2_ESM.zip › MetabolicTherapiesModel/Figures/Figures_Flux_noDrugs/figure_3.jpg]

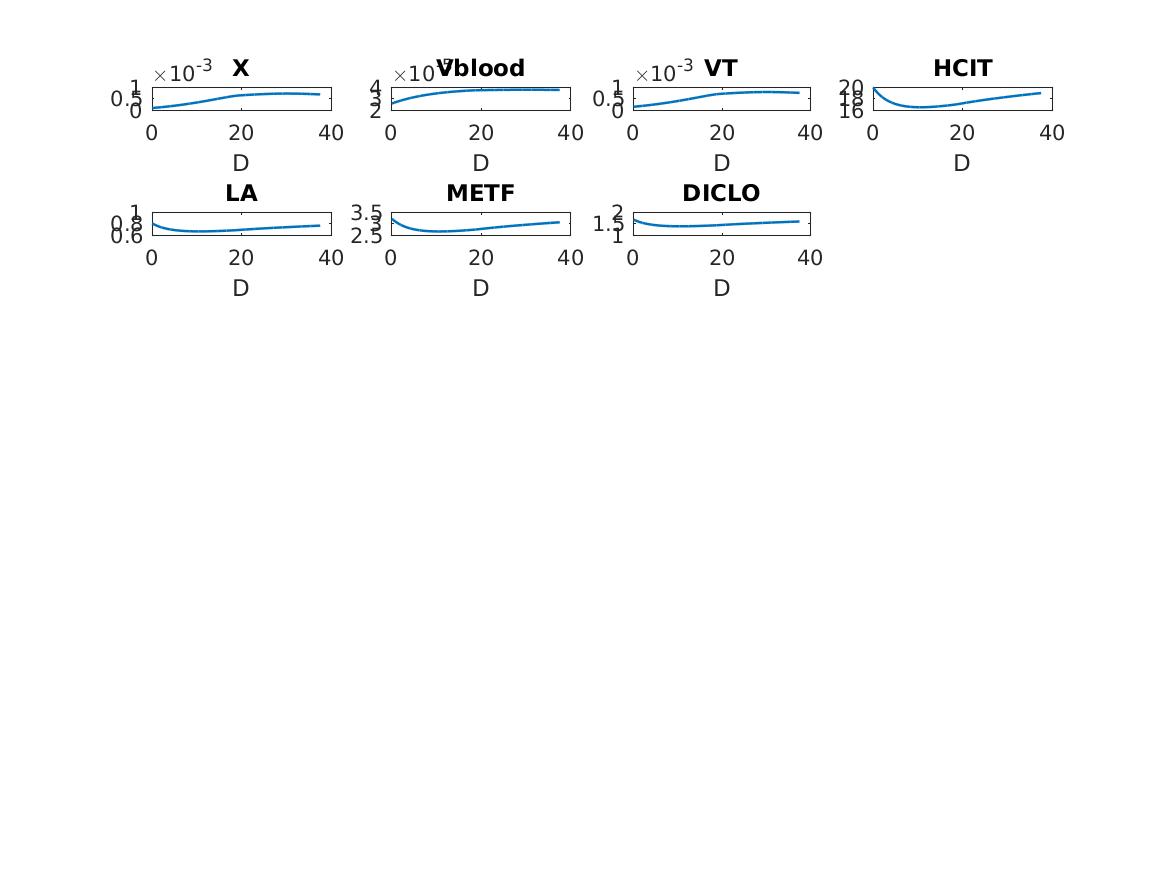

Supplement: Supplementary file 2 — Dataset 1 [file 41598_2019_39109_MOESM2_ESM.zip › MetabolicTherapiesModel/Figures/Figures_Metabolites_Drugs/figure_6.jpg]

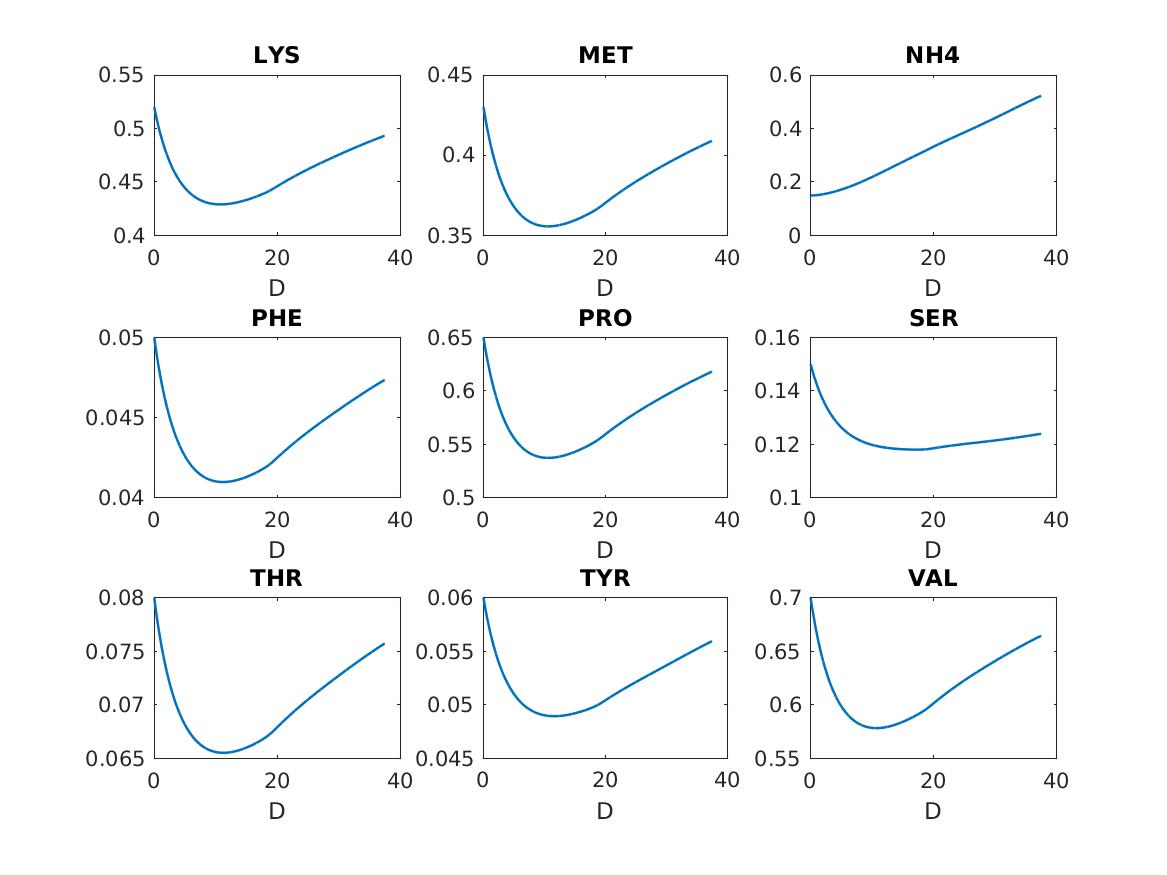

Supplement: Supplementary file 2 — Dataset 1 [file 41598_2019_39109_MOESM2_ESM.zip › MetabolicTherapiesModel/Figures/Figures_Metabolites_Drugs/figure_5.jpg]

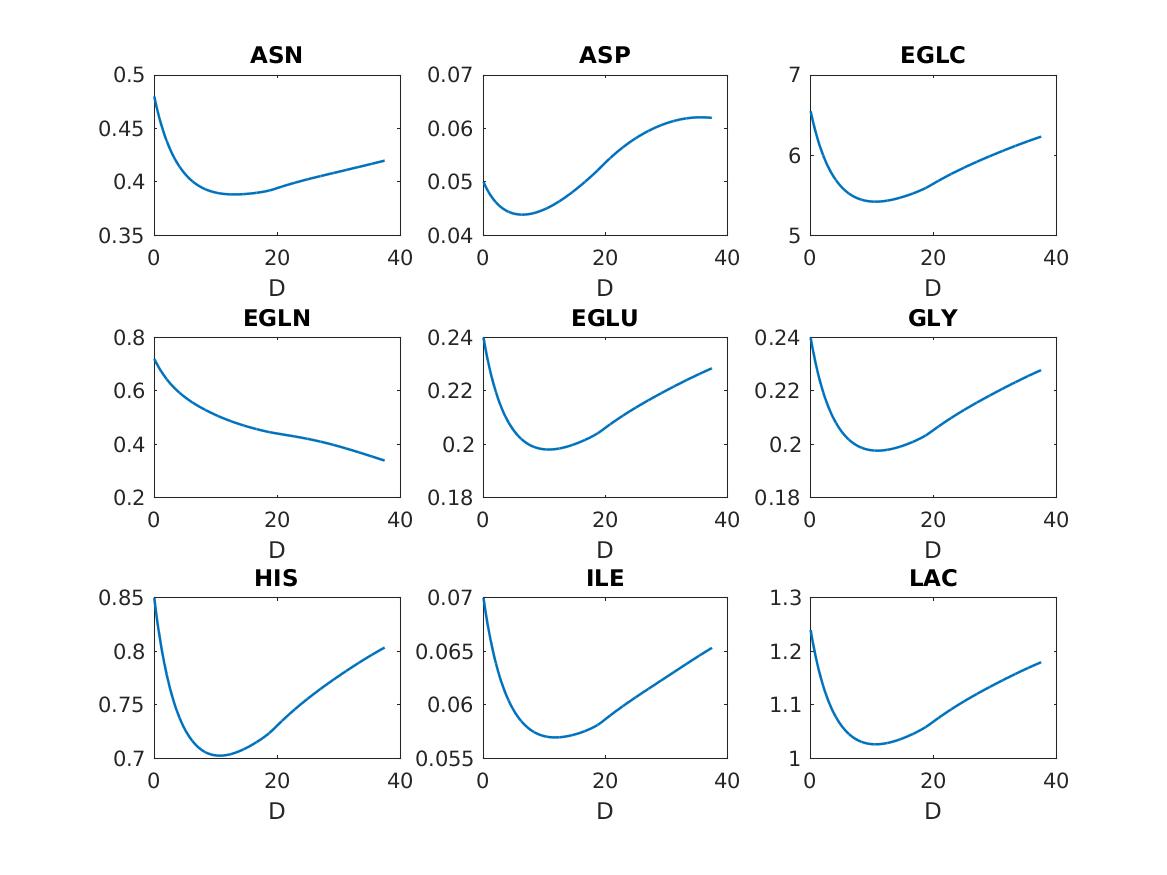

Supplement: Supplementary file 2 — Dataset 1 [file 41598_2019_39109_MOESM2_ESM.zip › MetabolicTherapiesModel/Figures/Figures_Metabolites_Drugs/figure_4.jpg]

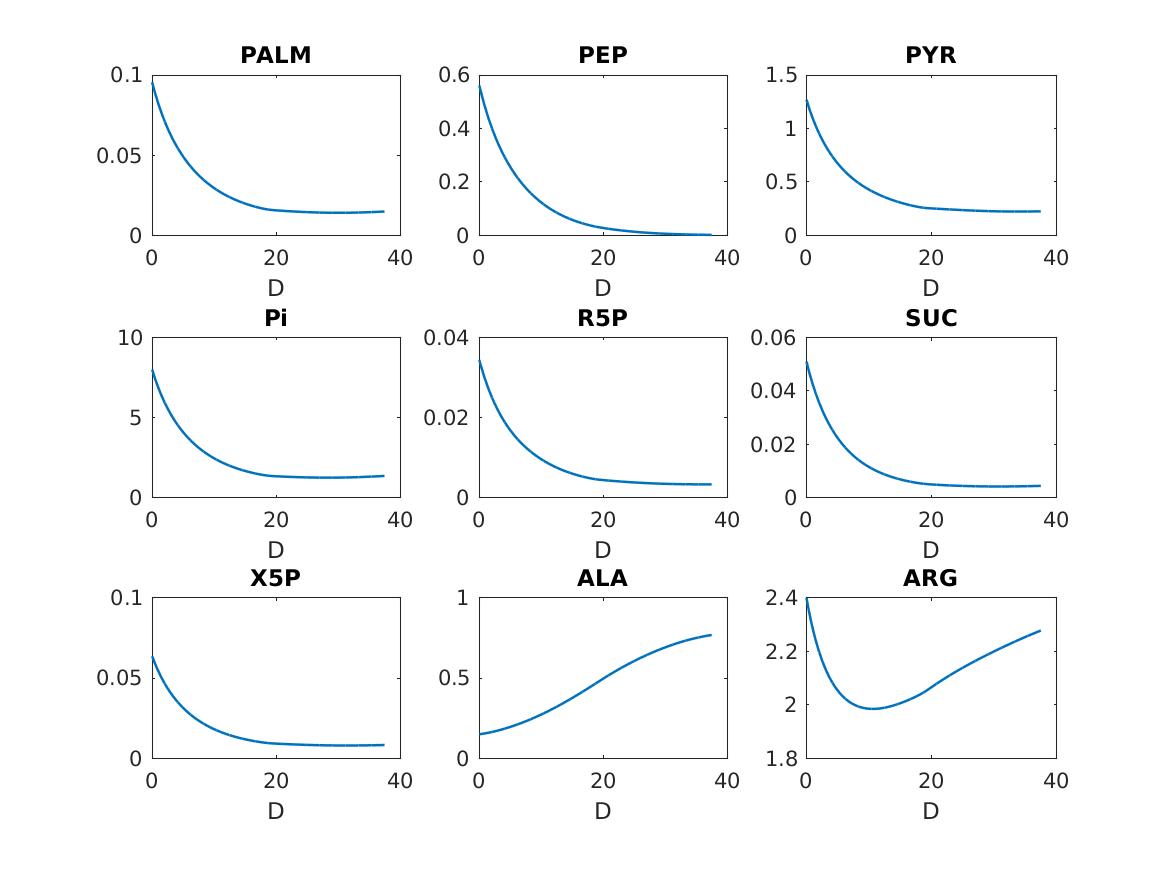

Supplement: Supplementary file 2 — Dataset 1 [file 41598_2019_39109_MOESM2_ESM.zip › MetabolicTherapiesModel/Figures/Figures_Metabolites_Drugs/figure_3.jpg]

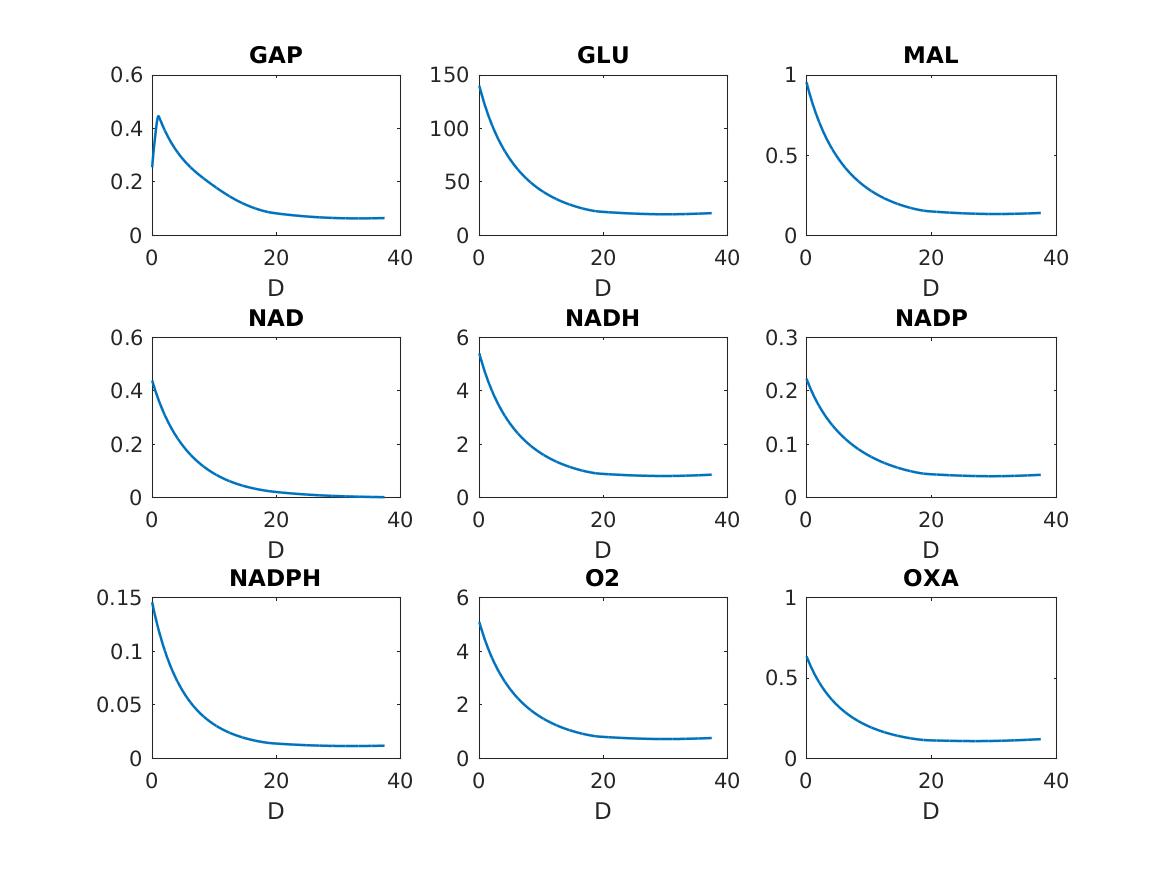

Supplement: Supplementary file 2 — Dataset 1 [file 41598_2019_39109_MOESM2_ESM.zip › MetabolicTherapiesModel/Figures/Figures_Metabolites_Drugs/figure_2.jpg]

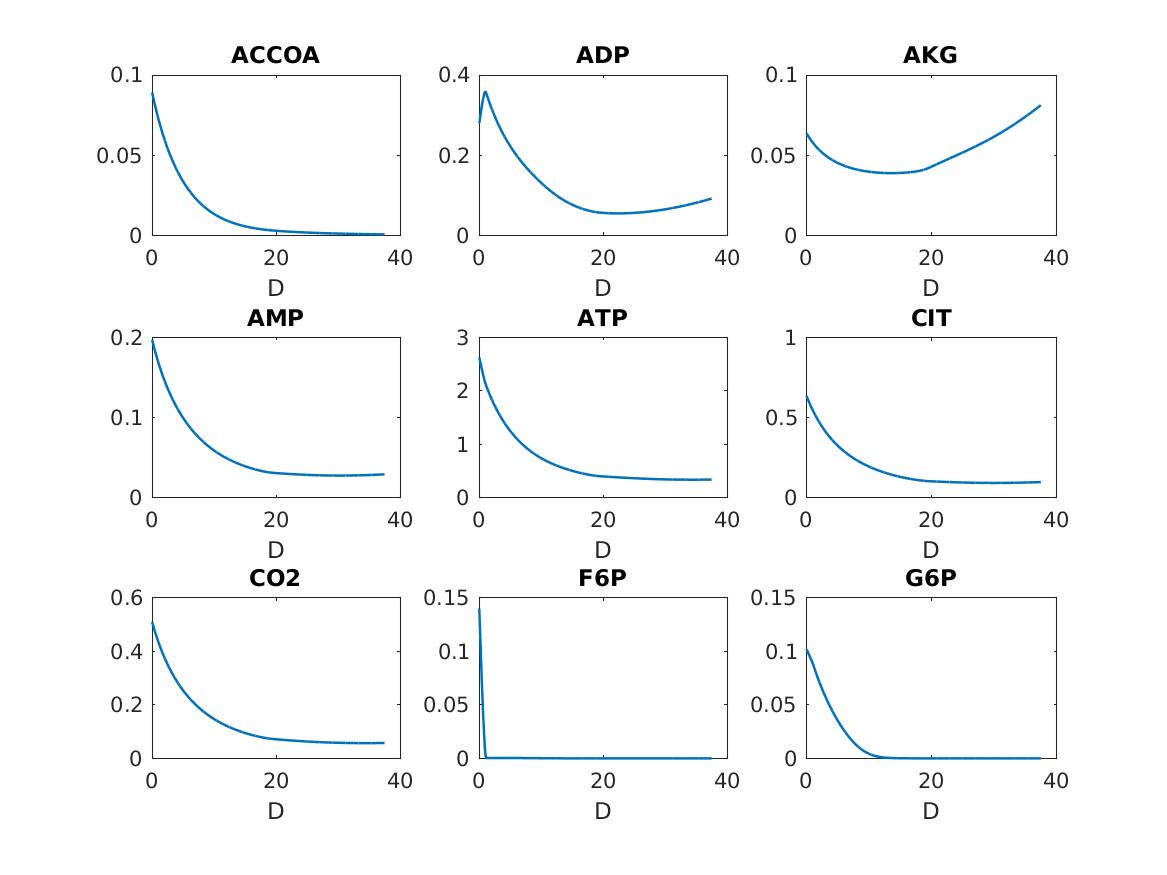

Supplement: Supplementary file 2 — Dataset 1 [file 41598_2019_39109_MOESM2_ESM.zip › MetabolicTherapiesModel/Figures/Figures_Metabolites_Drugs/figure_1.jpg]

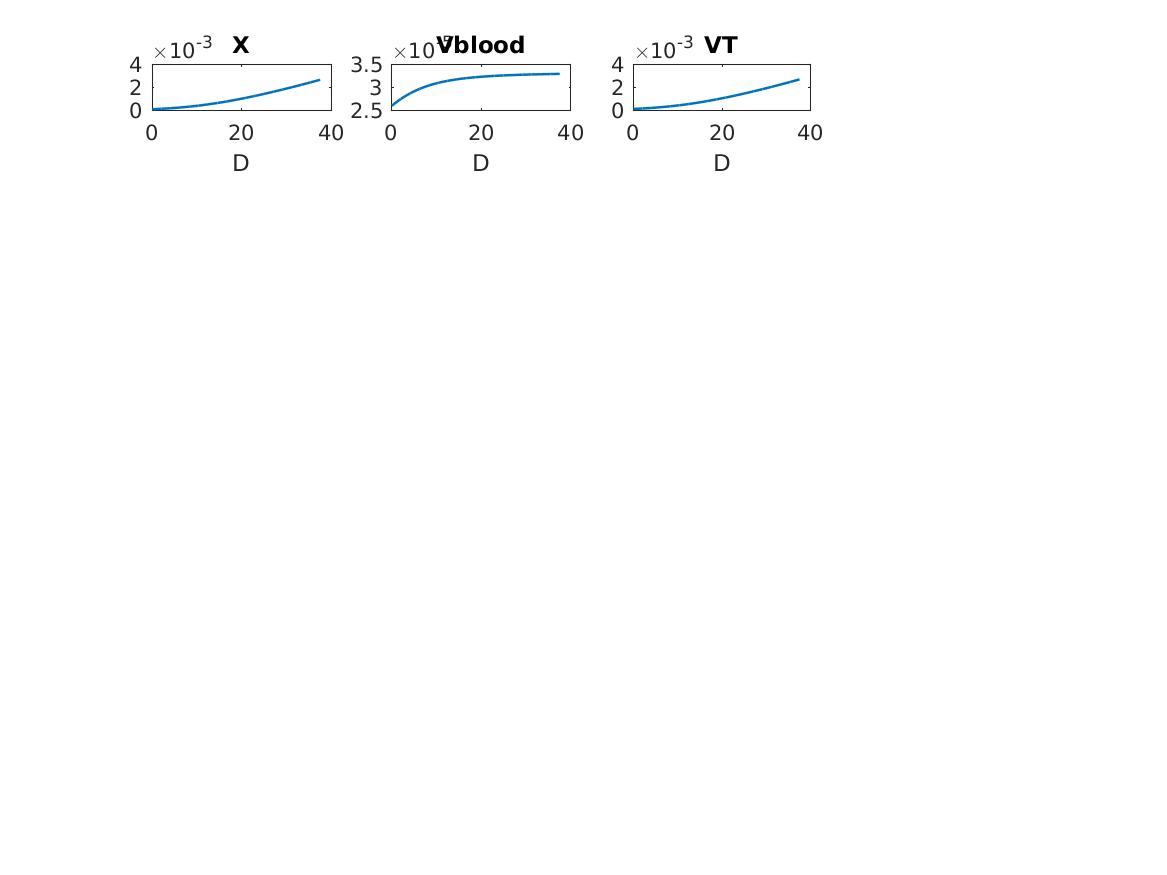

Supplement: Supplementary file 2 — Dataset 1 [file 41598_2019_39109_MOESM2_ESM.zip › MetabolicTherapiesModel/Figures/Figures_Metabolites_noDrugs/figure_6.jpg]

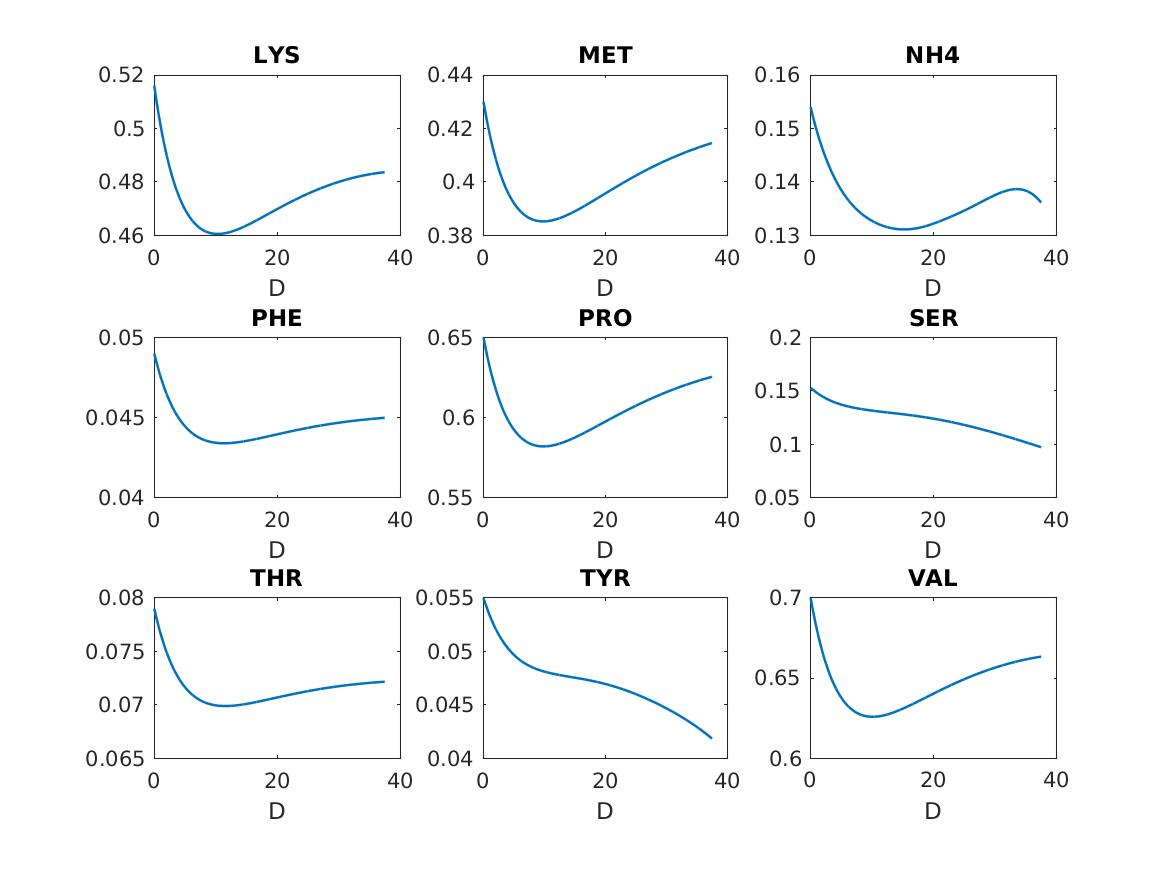

Supplement: Supplementary file 2 — Dataset 1 [file 41598_2019_39109_MOESM2_ESM.zip › MetabolicTherapiesModel/Figures/Figures_Metabolites_noDrugs/figure_5.jpg]

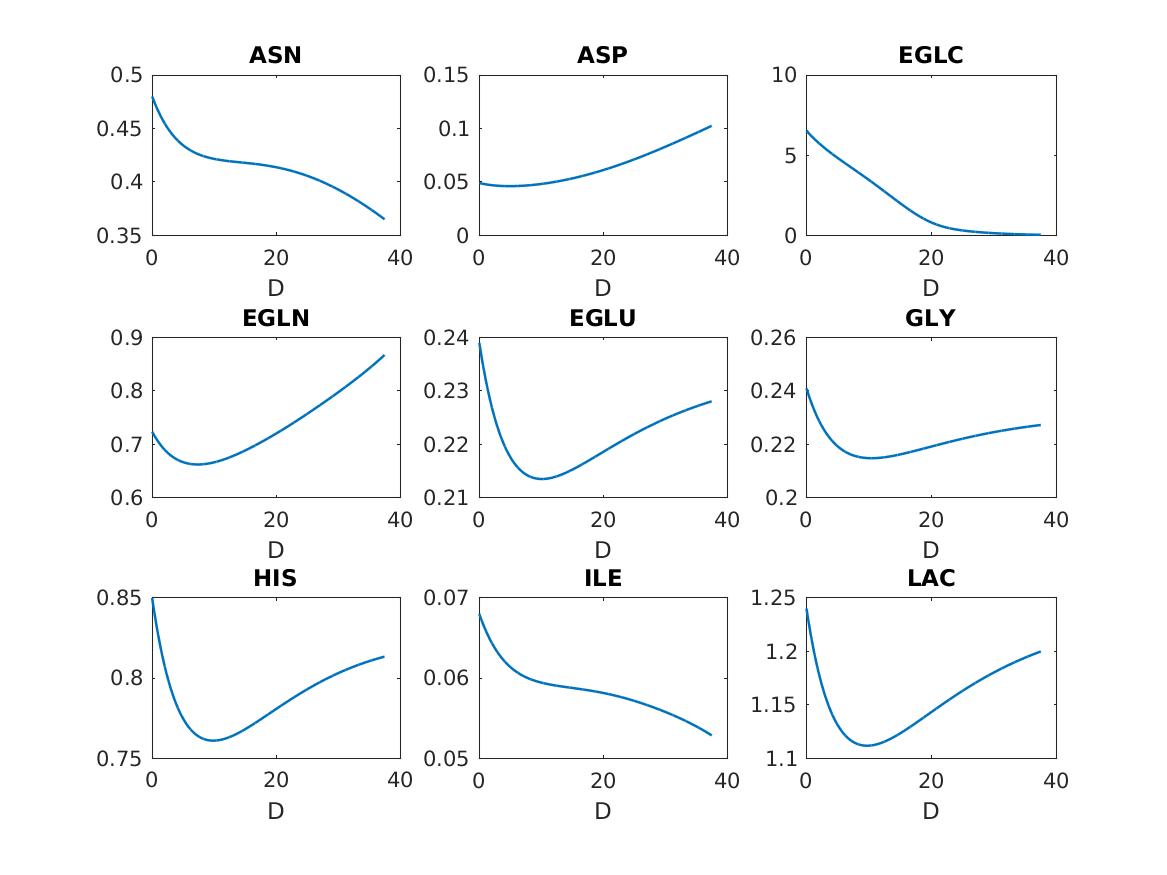

Supplement: Supplementary file 2 — Dataset 1 [file 41598_2019_39109_MOESM2_ESM.zip › MetabolicTherapiesModel/Figures/Figures_Metabolites_noDrugs/figure_4.jpg]

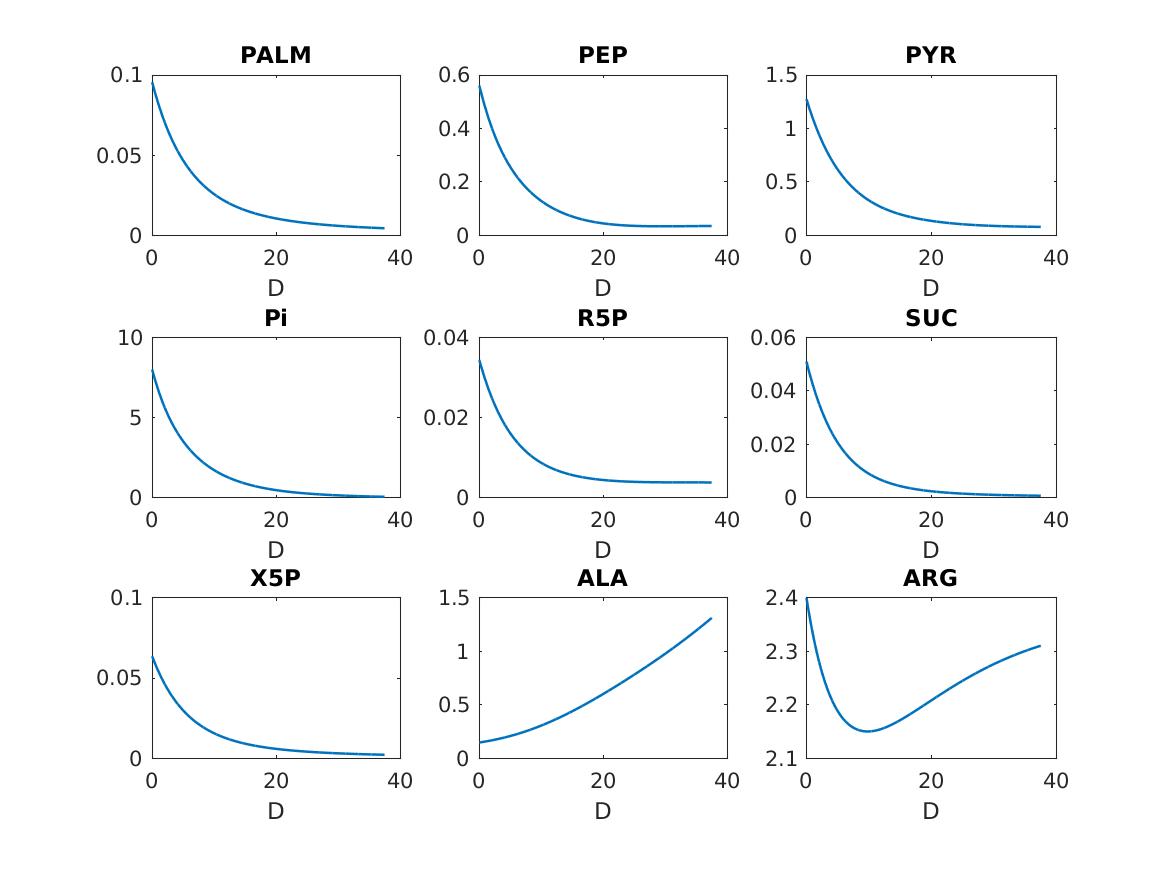

Supplement: Supplementary file 2 — Dataset 1 [file 41598_2019_39109_MOESM2_ESM.zip › MetabolicTherapiesModel/Figures/Figures_Metabolites_noDrugs/figure_3.jpg]

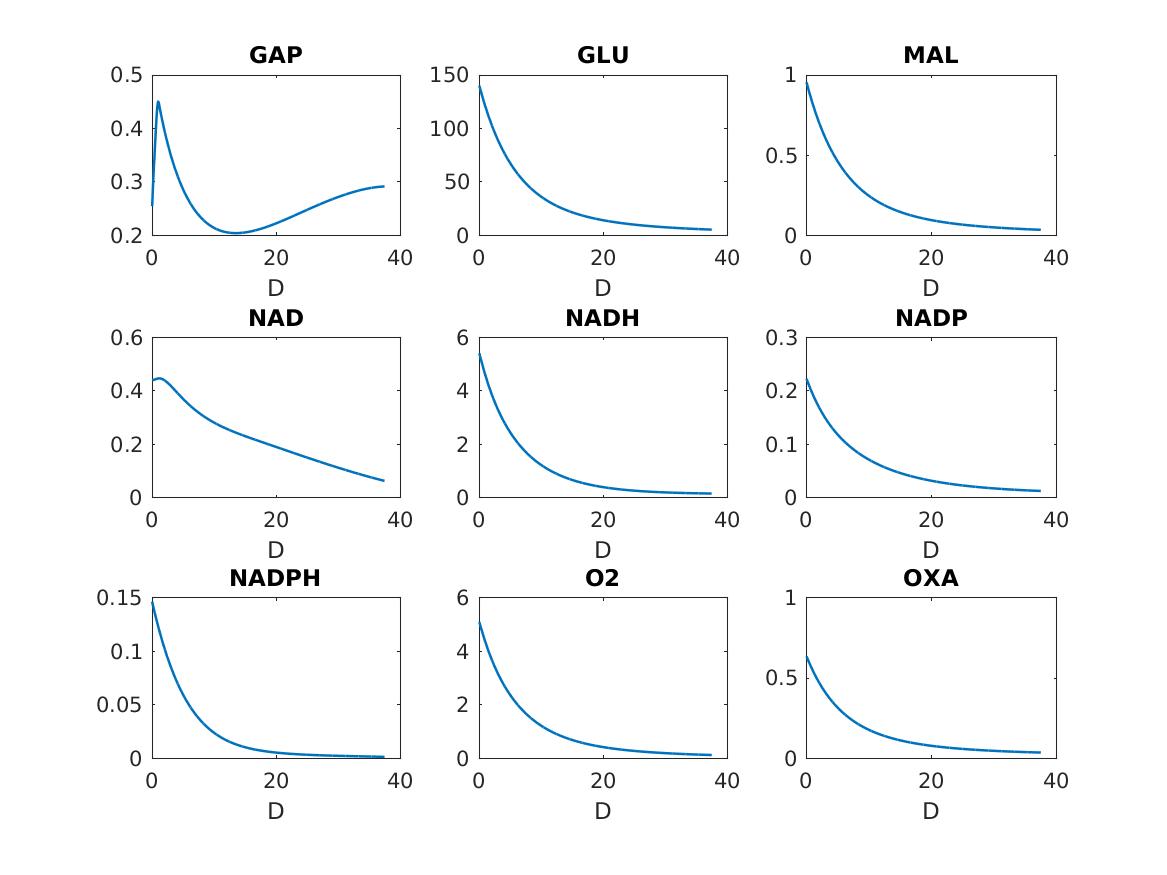

Supplement: Supplementary file 2 — Dataset 1 [file 41598_2019_39109_MOESM2_ESM.zip › MetabolicTherapiesModel/Figures/Figures_Metabolites_noDrugs/figure_2.jpg]

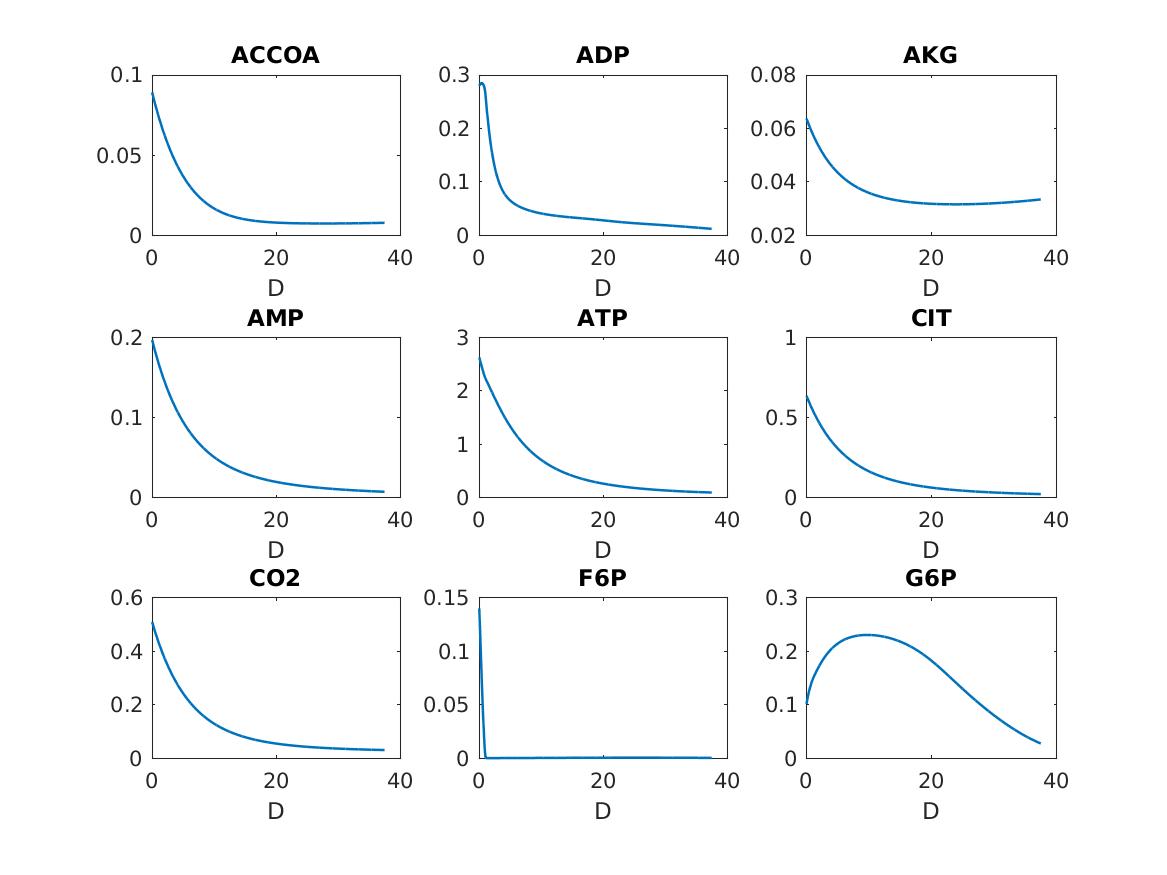

Supplement: Supplementary file 2 — Dataset 1 [file 41598_2019_39109_MOESM2_ESM.zip › MetabolicTherapiesModel/Figures/Figures_Metabolites_noDrugs/figure_1.jpg]
